# Exploration of metal‐free 2D electrocatalysts toward the oxygen electroreduction

**DOI:** 10.1002/EXP.20220174

**Published:** 2024-01-17

**Authors:** Joyjit Kundu, Taehyun Kwon, Kwangyeol Lee, Sang‐Il Choi

**Affiliations:** ^1^ Department of Chemistry and Green‐Nano Materials Research Center Kyungpook National University Daegu Republic of Korea; ^2^ Department of Chemistry and Research Institute of Basic Sciences Incheon National University Incheon Republic of Korea; ^3^ Department of Chemistry and Research Institute for Natural Sciences Korea University Seoul Republic of Korea

**Keywords:** 2D materials, electrocatalysts, non‐metal, oxygen reduction reaction

## Abstract

The advancement of economical and readily available electrocatalysts for the oxygen reduction reaction (ORR) holds paramount importance in the advancement of fuel cells and metal‐air batteries. Recently, 2D non‐metallic materials have obtained substantial attention as viable alternatives for ORR catalysts due to their manifold advantages, encompassing low cost, ample availability, substantial surface‐to‐volume ratio, high conductivity, exceptional durability, and competitive activity. The augmented ORR performances observed in metal‐free 2D materials typically arise from heteroatom doping, defects, or the formation of heterostructures. Here, the authors delve into the realm of electrocatalysts for the ORR, pivoting around metal‐free 2D materials. Initially, the merits of metal‐free 2D materials are explored and the reaction mechanism of the ORR is dissected. Subsequently, a comprehensive survey of diverse metal‐free 2D materials is presented, tracing their evolutionary journey from fundamental concepts to pragmatic applications in the context of ORR. Substantial importance is given on the exploration of various strategies for enhancing metal‐free 2D materials and assessing their impact on inherent material performance, including electronic properties. Finally, the challenges and future prospects that lie ahead for metal‐free 2D materials are underscored, as they aspire to serve as efficient ORR electrocatalysts.

## Graphical abstract

This review presents recent advances in metal‐free 2D materials for the oxygen reduction reaction (ORR), covering mechanistic insights, catalyst development, and performance enhancement. It also addresses challenges and future prospects for the development of high‐performance metal‐free 2D electrocatalysts toward the ORR.

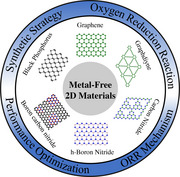

## 1 INTRODUCTION

The exploration of green and renewable energy generation systems such as fuel cells and metal‐air batteries has become a necessity in recent years due to the rapid growing issues such as climate change, environmental pollution, and energy security.^[^ ^1^, ^2^, ^3^, ^4^, ^5^, ^6^, ^7^ ^]^ To promote the performance of these energy systems, it is crucial to understand the fundamental principles of electrocatalysis.^[^ ^8^, ^9^, ^10^, ^11^, ^12^, ^13^, ^14^, ^15^ ^]^ Oxygen reduction reaction (ORR) is an electrochemical process in which O_2_ is reduced by four electrons at the cathode of fuel cells and metal‐air batteries,^[^ ^16^, ^17^, ^18^, ^19^ ^]^ and the sluggish ORR kinetics that limits the overall cell performance has been known as a major problem.^[^ ^20^, ^21^, ^22^, ^23^ ^]^

To tackle this issue, high‐performance Pt‐based catalysts have been adopted,^[^ ^24^, ^25^, ^26^, ^27^, ^28^, ^29^ ^]^ but their scarcity in the earth, high cost, and low stability restrict the large‐scale application.^[^ ^10^, ^30^, ^31^ ^]^ As an alternative, remarkable advances have been achieved in the development of non‐noble metal‐based catalysts, and some of them have demonstrated ORR activity similar to or even superior to the benchmark Pt catalyst.^[^ ^32^, ^33^, ^34^, ^35^, ^36^, ^37^ ^]^ However, non‐noble metal‐based catalysts are much less stable under high operating potentials during ORR. Apart from the development of non‐noble metal‐based catalysts, the recent discovery of metal‐free carbon‐based ORR catalysts has opened up new horizons.^[^ ^38^, ^39^, ^40^, ^41^, ^42^ ^]^ Metal‐free carbon‐based catalysts boast advantages in tolerance to the fuel, stable chemical structure, and good stability in alkaline media.^[^ ^43^, ^44^, ^45^ ^]^

During the ORR process, O_2_ molecules undergo adsorption and diffusion on the catalyst surface.^[^ ^1^, ^46^ ^]^ Moreover, a change in the catalyst morphology and surface structure often results in variations in active sites, controlling the performance of ORR.^[^ ^47^, ^48^ ^]^ 2D structured catalysts are highly appealing for ORR electrocatalysis due to their affordability, large surface area, exceptional physicochemical properties, superior electrical conductivity, high mechanical strength, and outstanding chemical stability.^[^ ^49^, ^50^, ^51^, ^52^ ^]^ Especially, the electronic structure of active sites and the surface geometric arrangements of 2D electrocatalysts play vital roles in controlling the energy barriers of the reactions, and thereby enhancing the intrinsic activity.^[^ ^53^, ^54^, ^55^, ^56^ ^]^ In this regard, metal‐free 2D electrocatalysts have received surging interest owing to their significantly large surface‐to‐volume ratio, allowing the maximum exposure of active sites, and enhanced ORR activity.

Apart from morphology, the surface defect is another factor controlling the number of active sites.^[^ ^57^, ^58^ ^]^ The abundance of exposed surface atoms on 2D materials facilitates the formation of defect structures, crystal twinning, and corrugated morphologies.^[^ ^59^ ^]^ These structural characteristics can significantly impact the material's intrinsic properties and enhance catalytic activities. The number of active sites on a 2D surface can be further increased by considering dopants, edges, twists, and size.^[^ ^59^, ^60^, ^61^ ^]^ Therefore, metal‐free 2D materials offer a versatile platform for tailoring properties to achieve desired functionalities, making it a highly active and dynamic field of research. However, the limited range of 2D materials and challenges in producing them on a large scale gravely hampered their practical application.

**FIGURE 1:**
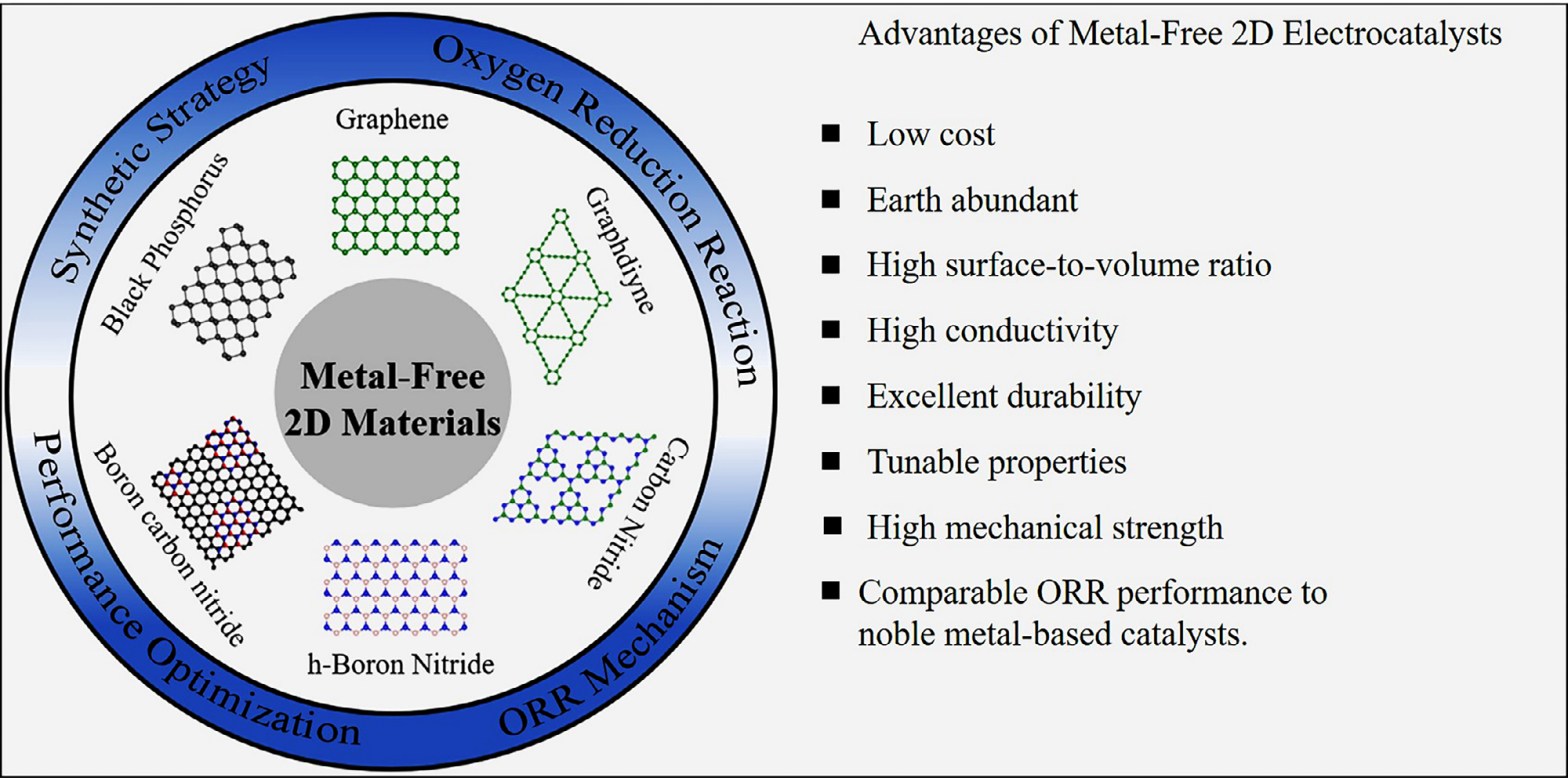
Summary of metal‐free 2D electrocatalysts for ORR.

Though there are some review articles on the non‐metallic materials for ORR, present articles particularly focused on the 2D structure of metal‐free nanomaterials for ORR. This review aims to comprehensively summarize recent developments in 2D metal‐free ORR catalysts and discuss the mechanistic understanding specific to them, as shown in Figure 1. First, we introduce the fundamental mechanisms of ORR, emphasizing the significance of ORR in fuel cells. Later, we discuss carbon‐based and non‐carbon‐based 2D materials and their ORR activity. We also include different factors, such as heteroatom doping, heterostructure formation, and generation of defects, which impact the ORR performance of the catalysts. Finally, the challenges and perspectives of this rapidly growing topic are discussed.

## 2 MECHANISTIC UNDERSTANDING OF THE ORR

### 2.1 A general ORR mechanism for metal‐based catalysts

It has been widely accepted that the cathodic ORR undergoes via either a four‐electron (direct) pathway or a series of two‐electron pathways. The reaction mechanism and the electron transfer process largely depend on the electrolytes (either acidic or alkaline). The overall pathways are as follows (Equations (1)−(4)), where SHE stands for standard hydrogen electrode:^[^ ^62^, ^63^ ^]^

Four‐electron pathway

(Alkaline)

(1) $$O_{2}+2H_{2}O+4e^{-}\to 4OH^{-}\;\left(E^{o}=0.401V_{\mathrm{SHE}}\right)$$

(Acidic)

(2) $$O_{2}+4H^{+}+4e^{-}\to 2H_{2}O\;\left(E^{o}=1.229V_{\mathrm{SHE}}\right)$$

Two‐electron pathway

(Alkaline)

(3a) $$O_{2}+2H_{2}O+2e^{-}\to {\mathrm{HO}}_{2}^{-}+OH^{-}\;\left(E^{o}=-0.076V_{\mathrm{SHE}}\right)$$

followed by further two‐electron reduction

(3b) $${\mathrm{HO}}_{2}^{-}+H_{2}O+2e^{-}\to 3OH^{-}\;\left(E^{o}=0.878V_{\mathrm{SHE}}\right)$$

or disproportionation

(3c) $$2{\mathrm{HO}}_{2}^{-}\to 2OH^{-}+O_{2}$$

(Acidic)

(4a) $$O_{2}+2H^{+}+2e^{-}\to H_{2}O_{2}\;\left(E^{o}=0.695V_{\mathrm{SHE}}\right)$$

followed by further two‐electron reduction

(4b) $$H_{2}O_{2}+2H^{+}+2e^{-}\to 2H_{2}O\;\left(E^{o}=1.776V_{\mathrm{SHE}}\right)$$

or disproportionation

(4c) $$2H_{2}O_{2}\to 2H_{2}O+O_{2}$$

For the purpose of the fuel cell or metal‐air battery applications, a direct four‐electron pathway is preferred to a series of indirect two‐electron pathways because the formation of intermediate H_2_O_2_ or HO_2_^–^ would reduce the overall number of transferred electrons. Moreover, versatile H_2_O_2_ and HO_2_^–^ would significantly degrade ionomers and electrocatalysts, reducing overall stability.^[^ ^5^, ^64^, ^65^ ^]^

For the direct four‐electron pathway of the ORR, the reaction proceeds (i) adsorption of O_2_ molecules, (ii) electron transfer to adsorbed O_2_, (iii) weakening and scission of O═O bonds, and (iv) desorption of products (H_2_O or OH^–^). Under alkaline electrolyte, the associative mechanism goes through the following steps (Equation (5)), where * represents a surface of electrocatalysts and *O_2_, *OOH, *OH, and *O represent adsorbed reaction intermediates:^[^ ^63^ ^]^

(5a)

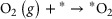



(5b)

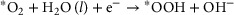



(5c)

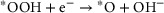



(5d)

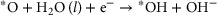



(5e)

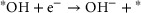

Competitive two‐electron mechanism

(5f)

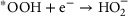




In acidic electrolytes, H_3_O^+^ rather than H_2_O acts as the proton donor, and the corresponding reaction steps are as follows:^[^ ^63^ ^]^

(6a)

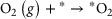



(6b)

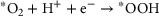



(6c)

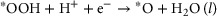



(6d)

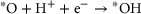



(6e)

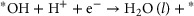




Competitive two‐electron mechanism

(6f)

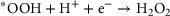




The selectivity of four‐ and two‐electron ORR is determined by the competitive reactions after forming the *OOH intermediate, whether the facilitating O─O bond scission occurs (Equations (5f) and (6f)).^[^ ^66^ ^]^ Conversely, the dissociative mechanism via direct cleavage of the O═O bond initiates with the following step (Equation (7)), followed by Equations (5d–e) or (6d–e) in alkaline or acidic media, respectively.^[^ ^62^ ^]^

(7a)

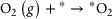



(7b)

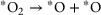




**FIGURE 2:**
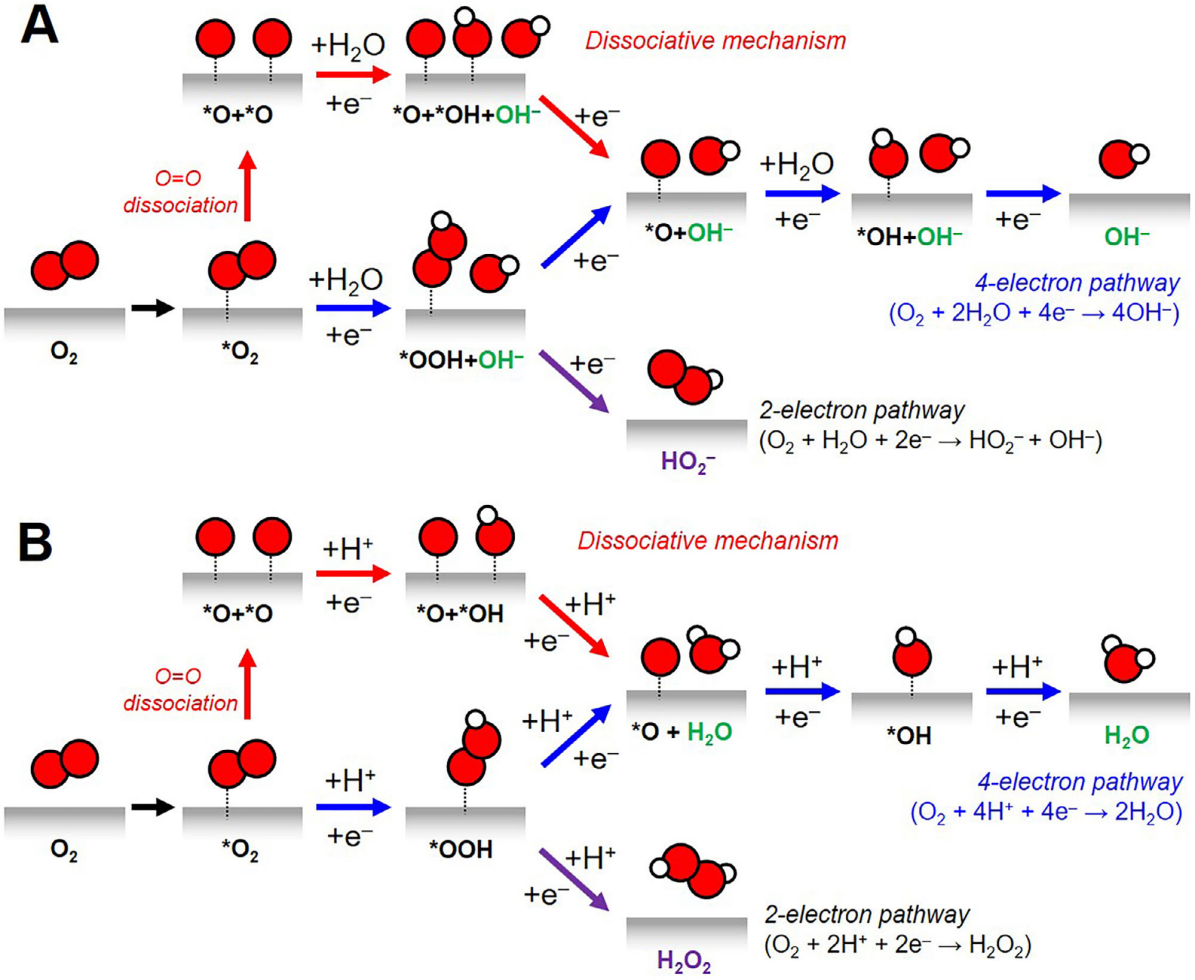
Schematic illustration of potential mechanisms of the ORR under (A) basic and (B) acidic conditions.

However, due to the higher bond dissociation energy of the O═O bond in O_2_ than O─O bond in superoxides, the associative mechanism is preferred to the dissociative mechanism.^[^ ^62^ ^]^ Overall plausible mechanisms of the ORR are summarized in Figure 2.

The theoretical descriptor for the ORR activity for metal‐based electrocatalysts has been widely explored based on the *d*‐band theory.^[^ ^67^, ^68^ ^]^ In 2004, Norskøv et al. employed density functional theory (DFT) and compared free energy landscapes for the binding of the ORR intermediates on the Pt(111) surface.^[^ ^69^ ^]^ The DFT study on the model Pt(111) surface suggested that the binding energies of *O, *OH, and *OOH intermediates are strongly correlated, which indicates scaling relations between *OOH and *OH or *OOH and *O.^[^ ^67^ ^]^ According to these scaling relations, the limiting potential for the ORR on the metal surface shows Sabatier volcano shape versus the difference of theoretical adsorption free energy of *OH intermediate (Δ*G*_OH_), which would be changed by the *d*‐band center of the metal surface.^[^ ^68^ ^]^ The commonly‐accepted rate‐determining step for the ORR is the desorption of *OH to form H_2_O or the adsorption of O_2_ to form *OOH.^[^ ^67^, ^70^ ^]^ Moreover, the selectivity of four‐ and two‐electron ORR is determined by the binding energies of *O and *OOH intermediates.^[^ ^63^, ^66^, ^71^ ^]^ The four‐electron pathway is chosen when the adsorption of *O intermediate from O─O bond dissociation is more favored than *OOH intermediate. On the other hand, more dominant adsorption of *OOH species over *O intermediate facilitates a two‐electron pathway over a four‐electron pathway because the *OOH intermediates are preserved with the suppression of O─O dissociation.^[^ ^66^, ^71^ ^]^

### 2.2 An ORR mechanism for metal‐free catalysts

For the metal‐free electrocatalysts, the overall ORR mechanism also involves the adsorption of O_2_ and the ORR intermediates, similar to the mechanism in metal‐based electrocatalysts. However, the ORR thermodynamics and kinetics could not be explained by the adsorption energies of reaction intermediates from the *d*‐band theory in the metal‐free electrocatalysts because they are generally composed of carbon. Moreover, pristine sp^2^ carbon without any defects or dopants is inactive to the adsorption of O_2_ and the ORR intermediates.^[^ ^62^, ^66^ ^]^ Therefore, the outer sphere electron‐transfer mechanism is suggested as the ORR mechanism in metal‐free, carbon‐based electrocatalysts.^[^ ^72^, ^73^, ^74^ ^]^ In this mechanism, O_2_ molecules do not directly interact with the surface of the electrocatalysts; rather, the electron transfer occurs through a solution‐phase mediator such as an alkali metal cation.^[^ ^75^, ^76^, ^77^ ^]^ During ORR, the solvated molecular O_2_ cluster, O_2_(H_2_O)*_n_*, weakly interacts with surface‐adsorbed hydroxyl species to promote the two‐electron pathway, indicating that the outer‐sphere electron transfer occurs with the two‐electron reduction.^[^ ^74^, ^78^ ^]^

Therefore, to improve the four‐electron reduction pathway, it is strongly required to regulate the energy states of basal carbon to promote the adsorption of O_2_ and the ORR intermediates. Particularly in metal‐free, carbon‐based electrocatalysts, hetero‐atom doped sites,^[^ ^79^, ^80^ ^]^ or defective sites^[^ ^81^, ^82^, ^83^ ^]^ exhibited lower free energy for the adsorption of O_2_ and the intermediates (*O, *OH, and *OOH) than sp^2^ carbon atoms in the basal plane. Several recent theoretical and experimental studies revealed that the adsorption free energy of O_2_ and the ORR intermediates are changed by the local electron density (charge distribution), the electronegativity of adjacent dopants, the electron configuration of dopants, and hybridization (sp^2^ or sp^3^).^[^ ^82^, ^83^, ^84^, ^85^, ^86^ ^]^ However, the exact reaction mechanism and the active sites in metal‐free electrocatalysts toward the ORR are still under debate, and therefore, further thorough investigation on both theoretical and experimental is strongly required.

## 3 CARBON‐BASED 2D ELECTROCATALYSTS FOR THE ORR

Carbon‐based ORR catalysts started to gain attention after the invention of N‐doped carbon nanotubes as an alternative to Pt catalysts.^[^ ^10^, ^38^, ^87^ ^]^ Since then, enormous progress has been attained in metal‐free 2D catalysts in the ORR process. In this section, we discuss carbon‐based 2D ORR catalysts such as graphene, graphyne, and graphitic carbon nitride.

### 3.1 Graphene and graphyne

**FIGURE 3:**
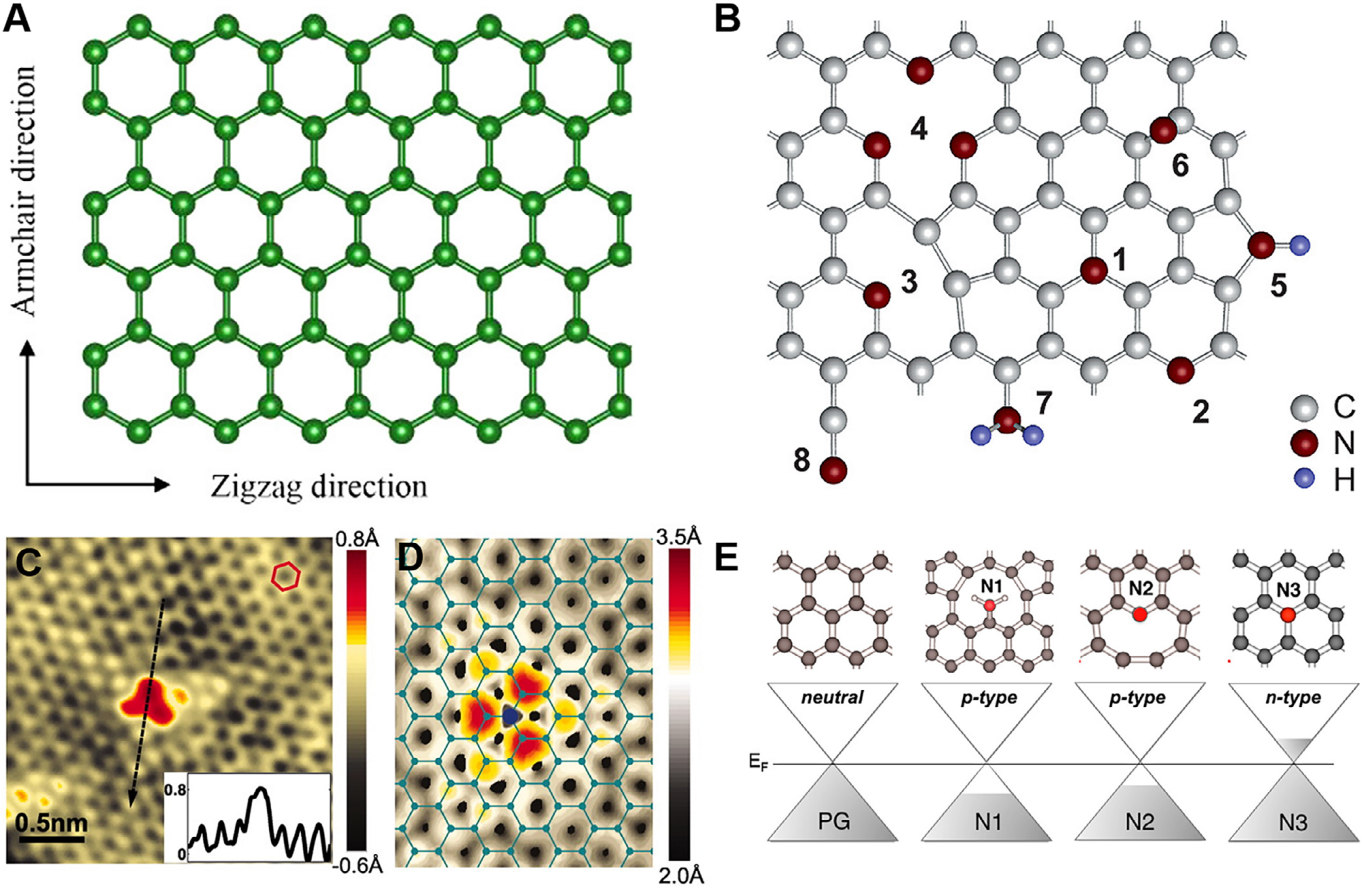
(A) Schematic molecular configuration of graphene. Reproduced with permission.^[^ ^1^ ^]^ Copyright 2018, American Chemical Society. (B) Different types of N impurities in graphene (1) substitutional or graphitic N, (2) pyridine‐like N, (3) single N pyridinic vacancy, (4) triple N pyridinic vacancy, (5) pyrrole‐like, (6) interstitial N or adatom, (7) amine, (8) nitrile. Reproduced with permission.^[^ ^90^ ^]^ Copyright 2011, American Chemical Society. (C) STM image of most common doping form observed on N‐doped graphene, corresponding to a single graphitic N dopant. The inset showing the atomic corrugation and apparent height of the dopant. (D) Simulated STM image of graphitic N dopant based on DFT calculations. Reproduced with permission.^[^ ^94^ ^]^ Copyright 2011, Science. (E) Computed doping effect of N dopant. Reprinted with permission.^[^ ^95^ ^]^ Copyright 2012, American Chemical Society.

Graphene is composed of hexagonally arranged sp^2^ carbon atoms, which resemble a honeycomb structure (Figure 3A). For electrocatalysis, the delocalized π bonding network within graphene could induce the endothermic adsorption of reaction intermediates.^[^ ^88^ ^]^ Therefore, the basal plane of graphene is electrocatalytically inactive. Defect engineering, heteroatom doping, and heterostructure formation can be conveniently employed to fine‐tune the electronic and surface properties of graphene. High electrical conductivity and large surface area are prerequisites for the successful electrocatalytic applications. As N exhibits higher electron negativity and one more valence electron than C, N has been the favorable choice to tune the overall electronic structure of graphene, to increase its conductivity, as well as to create active sites toward the ORR.^[^ ^89^ ^]^ As shown in Figure 3B, doping leads to the formation of different types of N doping such as graphitic N, pyrrolic N, pyridinic N, amide N, and N oxides.^[^ ^90^ ^]^ The planar structure of graphene can be disrupted by the inclusion of an sp^3^‐bonded pyrrolic N atom. However, pyridinic N and graphitic N do not significantly affect the structure of graphene as the C─N bond length is very similar to the C─C bond length.^[^ ^91^ ^]^ Furthermore, pyridinic N has demonstrated excellent stability when a single vacancy is present. Interestingly, both Stone–Wales and vacancy defects presented in graphitic N and pyridinic N promoted the ORR performances.^[^ ^92^, ^93^ ^]^

Scanning tunnelling microscopy (STM) analysis can reveal the exact type of N doping in the graphene. Figure 3C,D show the STM and simulated STM images, respectively.^[^ ^94^ ^]^ Investigating the authentic catalytic site within N‐doped graphene holds significance due to the ambiguity surrounding whether the pyridinic N or graphitic N is the true active site. Anderson and co‐workers discovered that the graphitic N, which is far from the sheet edge, is more active than N atoms close to the edge.^[^ ^96^ ^]^ Miyata and co‐workers also reported that graphene with graphitic N performs better ORR activity than that with pyridinic N.^[^ ^97^ ^]^ In another work, Miyata and co‐workers showed that graphitic N atoms adjacent to the zigzag edge of graphene enhance the process of adsorption of O_2_ and subsequent ORR, resulting in the conversion of two molecules of H_2_O with a low activation barrier of approximately 5 kcal mol^‐1^.^[^ ^98^ ^]^ However, they suggested that the graphitic N atoms located on the inner side of the graphene sheet do not take part in the ORR. The conductive properties of N‐doped graphene are also influenced by its configuration. Every N atom forms three σ bonds with neighboring carbon atoms in graphitic N (N3) (Figure 3E).^[^ ^95^ ^]^ Additionally, one of the nitrogen's valence electrons forms a π bond with the neighboring carbon atoms, while the fifth electron contributes in the π* state of the junction band. The π network of the graphene lattice can receive 0.5 electrons from each N3. In contrast, pyridinic N (N2), and nitrilic N (N1) have the opposite electronic effect. They withdraw electrons from the π network of the graphene lattice.

**FIGURE 4:**
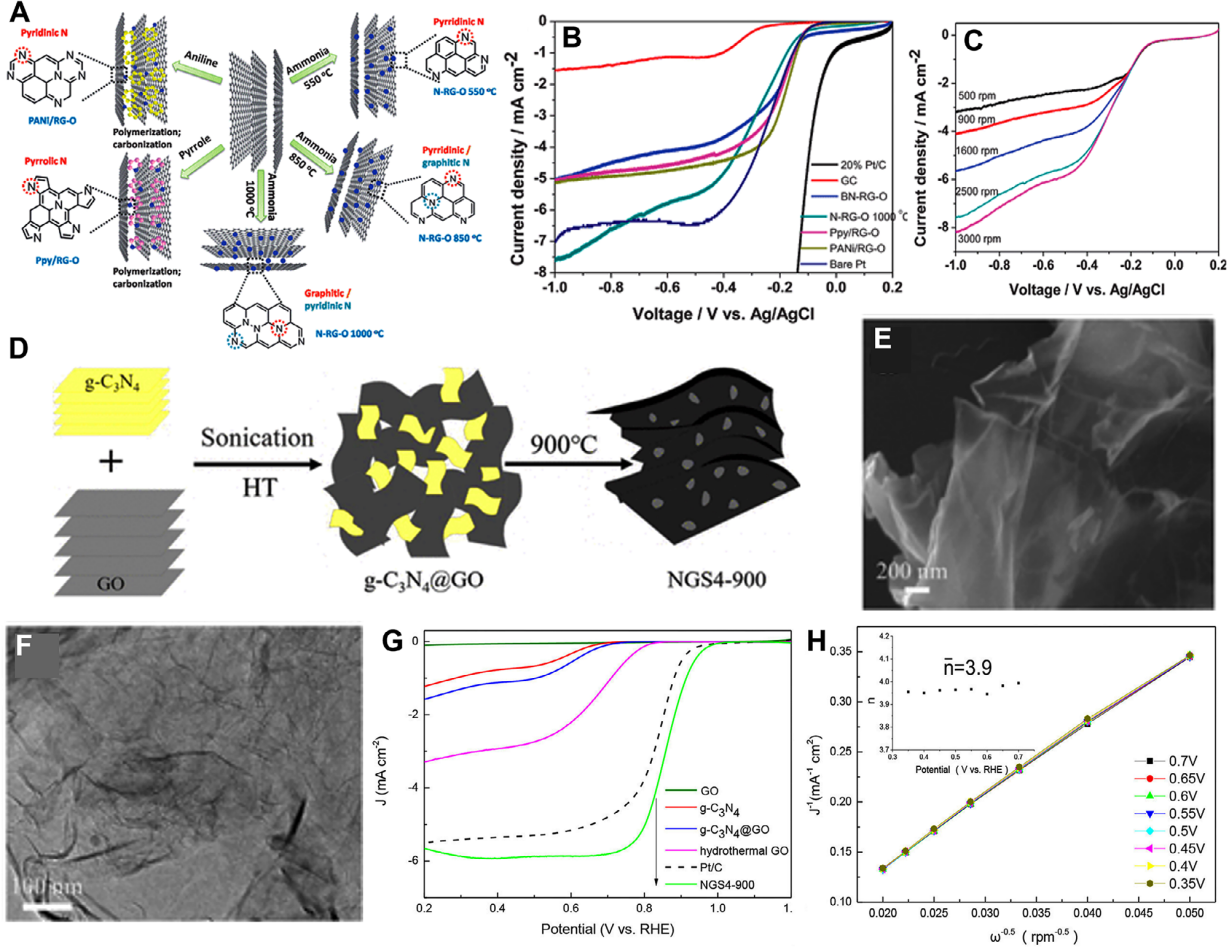
(A) Schematic representation of N‐doped graphene synthesis with different N states. (B) Linear sweep voltammetry (LSV) plot of N‐RG‐O 1000°C, bare GC electrode, PANi/RG‐O, Ppy/RG‐O, BN‐RG‐O, bare Pt electrode, and 20% Pt/C in O_2_ saturated 0.1 m KOH solution (scan rate: 10 mV s^‐1^, rotation rate: 2500 rpm). (C) LSV of N‐RG‐O 1000°C with a rotation rate from 500 to 3000 rpm. Reproduced with permission.^[^ ^101^ ^]^ Copyright 2012, Royal Society of Chemistry. (D) Schematic representation of the synthesis process of NGS4−900. TEM images of (E) g‐C_3_N_4_@GO and (F) NGS4−900. (G) LSV plots of GO, g‐C_3_N_4_, g‐C_3_N_4_@GO, hydrothermal GO, Pt/C, and NGS4−900 obtained on 1600 rpm RDE in O_2_ saturated 0.1 m KOH with 10 mV s^‐1^. (H) K–L plots of NGS4−900 at different potentials. Reproduced with permission.^[^ ^102^ ^]^ Copyright 2018, American Chemical Society.

Lazaro and co‐workers prepared N‐doped graphene by annealing graphene and urea at different temperatures ranging from 500–800°C.^[^ ^99^ ^]^ Lower temperature synthesis (500°C and 600°C) produced a small amount of carbon nitride (CN) along with N‐doped graphene, while higher temperatures led to higher purity of N‐doped graphene. It was observed that CN does not show promising ORR performance as a catalyst. A uniform 2D laminar structure was formed in all the cases. The N‐doped graphene synthesized at 800°C, which lacks CN, showed the highest ORR performance among all samples, with a halfwave potential (*E*_1/2_) of 0.76 V versus RHE, similar to commercial Pt/C. Guo and co‐workers adopted a pyrolysis process to synthesize N‐doped graphene, taking polyaniline as a nitrogen source.^[^ ^100^ ^]^ A typical 2D graphene morphology was generated with stacked and interlaced ultrathin nanosheets. In addition, a large number of pores were observed, which are believed to play a significant role in transporting the electron, thus improving the ORR activity. This N‐rich graphene showed an onset potential (*E*_onset_) of 0.99 V versus RHE and *E*_1/2_ of 0.84 V versus RHE. Ruoff and co‐workers investigated the impact of N precursor and temperature on the performance of the ORR.^[^ ^101^ ^]^ Figure 4A shows a schematic for the synthesis process for different N‐doped reduced graphene oxide (N‐RG‐O). The adoption of nitrogen atom‐containing aniline, pyrrole, and ammonia gas as precursors gave rise to the N‐doping of carbon materials. Aniline precursor created pyridinic N‐doped RGO, whereas pyrrole created pyrrolic N‐doped RGO. In the case of ammonia, the type of N‐doping was determined by the annealing temperature. At 550°C, only pyridinic N‐doped RGO was obtained, whereas at 850°C, a small amount of graphitic N‐doped RGO was formed along with pyridinic N‐doped RGO. In the case of 1000°C, mostly graphitic N‐doped RGO was produced with a small amount of pyridinic N‐doped RGO. Graphitic N containing RG‐O prepared at 1000°C (N‐RG‐O 1000°C) showed higher ORR performance than pyridinic N and pyrrolic N species (Figure 4B,C). The 2D structure of both N‐RG‐O 850°C and N‐RG‐O 1000°C have more exposed edges compared to pristine G‐O. It is observed that the total percentage of N in the system does not affect the ORR performance. Graphitic N controls the limiting diffusion current (*J*_L_), whereas pyridinic N controls the *E*_onset_. In all these cases, graphitic N is believed to play a major role in enhancing ORR performance.

Gupta and co‐workers reported that solvent treatment could alter the ORR performance of graphene oxide (GO).^[^ ^102^ ^]^ The solvent treatment was performed before the N doping process. Different solvents, such as water, ethanol, toluene, pyridine, and diethyl ether, were utilized for this treatment. They prepared an N‐doped GO catalyst in the presence of ammonia at 850°C. The ORR activity was more significantly affected in cyclic solvents such as toluene and pyridine than in non‐cyclic solvents like water, ethanol, and diethyl ether. In the case of water, *E*_1/2_ was 0.77 V versus RHE, which changed to 0.81 V in ethanol and diethyl ether. *E*_1/2_ increased to 0.80 V and 0.84 V versus RHE in the case of pyridine and toluene, respectively. Samples treated with pyridine and diethyl ether exhibited superior selectivity and performance in an alkaline medium. Chen and co‐workers prepared N‐doped graphene nanosheets (NGS4‐900) by pyrolysis (900°C) of g‐C_3_N_4_@GO gel‐like hybrid.^[^ ^103^ ^]^ First, the g‐C_3_N_4_@GO was prepared via a hydrothermal method by exfoliating g‐C_3_N_4_ sheets on GO sheets, which resulted in the 3D hybrid film structure. Then the g‐C_3_N_4_@GO was annealed at the temperature range of 700–1000°C under vacuum for 1 h. During the thermal expansion process, GOs are typically assembled into a fluffy porous 3D structure. This design improved the ease of accessing exfoliated g‐C_3_N_4_ sheets and promoted more efficient interactions between the two materials. During the annealing process of the g‐C_3_N_4_@GO composite, uniform distribution of nitrogen atoms occurred within the graphene matrix. Figure 4D shows a schematic diagram of the synthesis process. TEM images of g‐C_3_N_4_@GO and NGS4−900 are shown in Figure 4E,F, where ultra‐thin 2D morphology of NGS4‐900 is observed. The NGS4−900 showed a higher ORR activity than pure GO, g‐C_3_N_4_, and g‐C_3_N_4_@GO due to the doping of N atoms in the pyrolysis process, which forms abundant defect structure in the edge of graphene skeleton (Figure 4G). NGS4−900 exhibited *E*_onset_ (0.984 V), *E*_1/2_ (0.859 V), and *J*_L_ (5.98 mA cm^‐^^2^), which were higher than those of commercial Pt/C catalyst (0.971 V, 0.848 V, and 5.41 mA cm^‐^^2^), respectively. The calculated number of electrons per O_2_ molecule was 3.9, suggesting a four‐electron reaction pathway (Figure 4H). However, it still is difficult to conclude which, graphitic N or pyridinic N, is more responsible for the enhanced ORR performance.

In addition to N, elements from the p‐block like B, S, and P can be incorporated into the graphene matrix to enhance the ORR performance. This doping can be done either by chemical or physical approaches. As with N, the difference in size and electronegativity of these atoms to C can cause electron modulation, resulting in changes in the electronic properties of pristine graphene. The common precursors to dope these elements are boric acid or boron oxide for B; CS_2_, phenyl disulfide, or benzyl disulfide for S; triphenylphosphine for P.^[^ ^1^ ^]^ Theoretical calculations indicated that among the potential heteroatom dopants, B notably enhances the local spin density on the basal plane of graphene. This results in enhanced oxygen adsorption kinetics due to the presence of inherent acidic B‐C active sites, which promote hydroxyl adsorption.^[^ ^104^, ^105^ ^]^ Considerable electronegativity difference between B (2.04) and C (2.55) is the main reason behind this phenomenon. As a result, the paired covalent electrons of the B─C bonds experience a slight polarization, resulting in the formation of a localized positive charge density on the B atoms. These sites behave as strong acidic defects, providing better adsorption sites for O_2_ and its intermediates.^[^ ^106^ ^]^ The efficiency of electron transfer from the catalytic sites to the reaction intermediates is a pivotal factor that impacts the electrocatalytic activity of B‐doped graphene. It has been reported that during the ORR transfer process, the B sites behave as electron donors, where p‐electrons of graphene form a bonding p_z_ orbital that includes an unpaired electron.^[^ ^106^, ^107^, ^108^ ^]^ Incorporating B atoms into the graphene lattice is a challenging process, mainly because of the exceptional stability of the C─C bonds. However, because of their comparable atomic dimensions, B can replace C in the graphene lattice.^[^ ^108^, ^109^, ^110^, ^111^ ^]^

**FIGURE 5:**
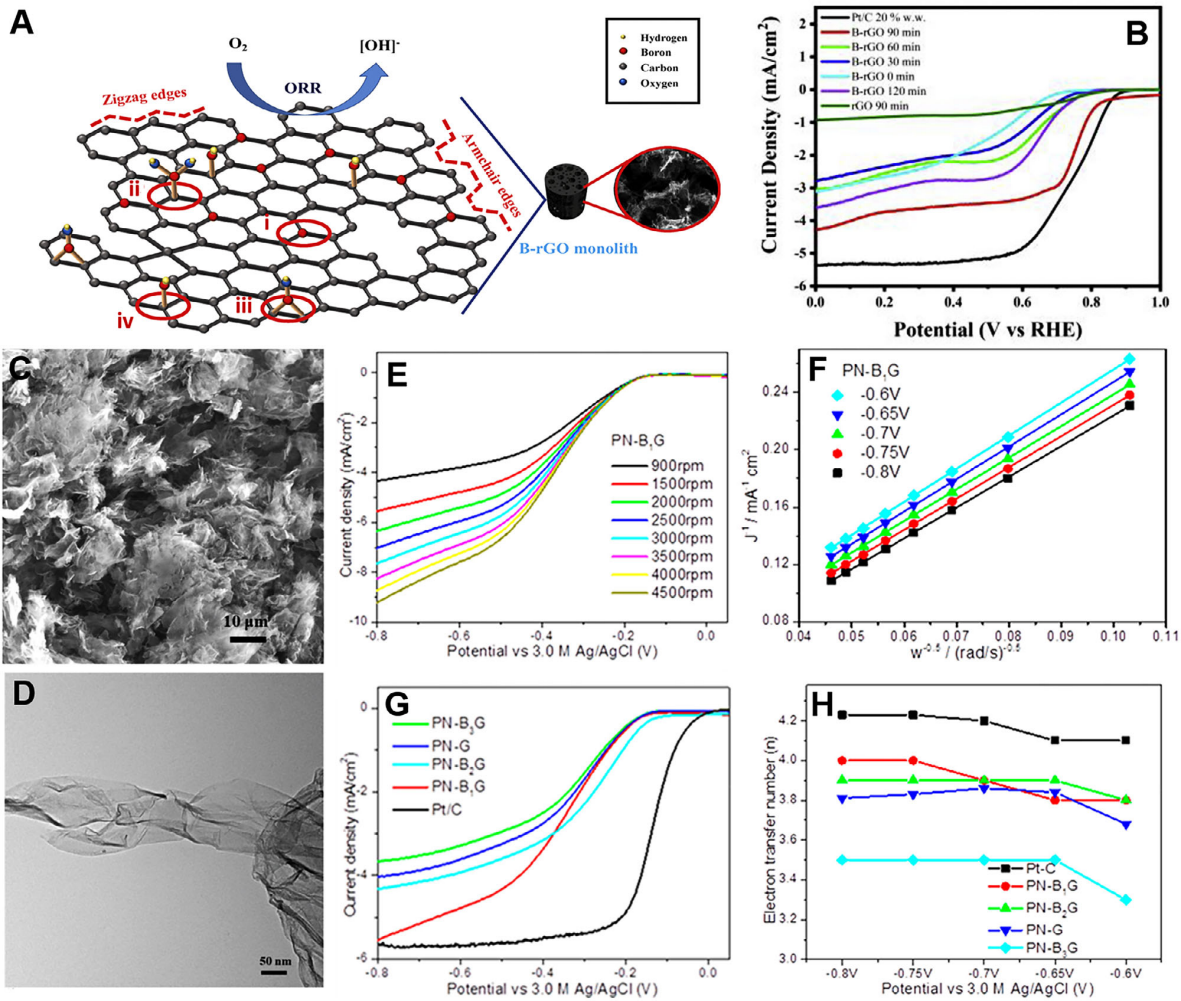
(A) Schematic representation of (i) B substitutional doping on graphene lattice, (ii) boronic, (iii) borinic, (iv) organo‐borane, and typical defects found in B‐doped rGO. (B) LSV plots of B‐doped rGO samples in 0.1 m KOH solution. Reproduced with permission.^[^ ^112^ ^]^ Copyright 2022, Elsevier. (C) FESEM and (D) TEM images of B_1_G. (E) LSV of PN‐B_1_G sample measured in an O_2_ saturated 0.1 m KOH solution with different rotation speeds at a scan rate of 5 mV s^‐1^. (F) K–L plots of PN‐B_1_G at different potentials. (G) LSV curves of different PN‐BG samples and Pt/C with a rotation speed of 1500 rpm at a scan rate of 5 mV s^‐1^. (H) Electron‐transfer number of different PN‐BG samples and Pt/C, calculated from the K–L equation. Reproduced with permission.^[^ ^113^ ^]^ Copyright 2020, American Chemical Society.

Four different kinds of B doping are possible: substitutional, boronic, borinic, and organo‐borane (Figure 5A).^[^ ^112^ ^]^ In the case of boronic doping, a carbon atom is attached to the B(OH)_2_ group, while in the case of borinic doping, two carbon atoms are connected to the B(OH) group. In the case of organo‐borane, the BH_2_ group is attached to the carbon atom of the graphene layer. The substitutional B doping has been predominantly attributed to electrocatalytic activity in most cases.^[^ ^108^, ^109^, ^110^, ^111^ ^]^ Sobrido and co‐workers proposed the increased B doping level by applying ultrasonication before hydrothermal treatment, leading to a higher ORR activity.^[^ ^112^ ^]^ They first sonicated GO solution for different time durations. Then, they added H_3_BO_3_ to the solution and heated the mixture in an autoclave at 180°C for 12 h. It is observed that the sample prepared with 90 min of ultrasonication showed a large surface area (438 m^2^ g^‐1^) compared to samples prepared with 60 min (301 m^2^ g^‐1^) and 30 min (211 m^2^ g^‐1^) of ultrasonication. It is also observed that the pore size of the B‐doped reduced graphene oxide (rGO) decreases with the ultrasonication time. B‐doped rGO prepared with 90 min of ultrasonication showed *E*_onset_ of 0.833 V versus RHE, which is 67 mV lower than the commercial Pt/C (Figure 5B). It also exhibits *J*_L_ of 4.1 mA cm^‐^^2^ (at 0.5 V vs RHE) and *E*_1/2_ of 0.671 V versus RHE. B‐doped rGO prepared in 90 min showed a calculated electron transfer number (*n*) of 3.7 under 0.5 V versus RHE, suggesting a four‐electron pathway. The higher performance of B‐rGO was attributed to B‐substitution doping, defect concentration, and surface area. Wang and co‐workers improved the activity of B‐doped graphene (BG) by depositing polynitrogen (N_8_^−^, PN).^[^ ^113^ ^]^ NaBH_4_ solution was added to the colloidal GO solution, which was heated in a Teflon‐lined stainless steel autoclave. By controlling the amount of initial boron precursor, three samples (B*_X_* G, *X* = 1, 2, 3) containing different amounts of B were prepared in the first step using a hydrothermal treatment method. Figure 5C,D shows the FESEM and TEM images of the B_1_G sample, confirming the 2D structure. PN‐BG was prepared using an electrochemical deposition technique to deposit N_8_^−^ on BG. A larger amount of N_8_^−^ were deposited on B_1_G compared to pure graphene. B doping enhanced the charge transfer from BG to PN, forming more PN on BG. PN‐B_1_G, which had a boron content of 1.23%, showed the highest *J*_L_ compared to PN‐B_2_G (2.01%), PN‐B_3_G (2.78%), and PN‐G (0%) (Figure 5E,G). The calculated electron transfer number for PN‐B_1_G was in the range of 3.8–4.0, suggesting a 4‐electron transfer process in ORR (Figure 5F,H). Larger amount of N_8_^−^ species were observed on B_1_G, which provided more active sites for ORR, leading to superior ORR performance.

**FIGURE 6:**
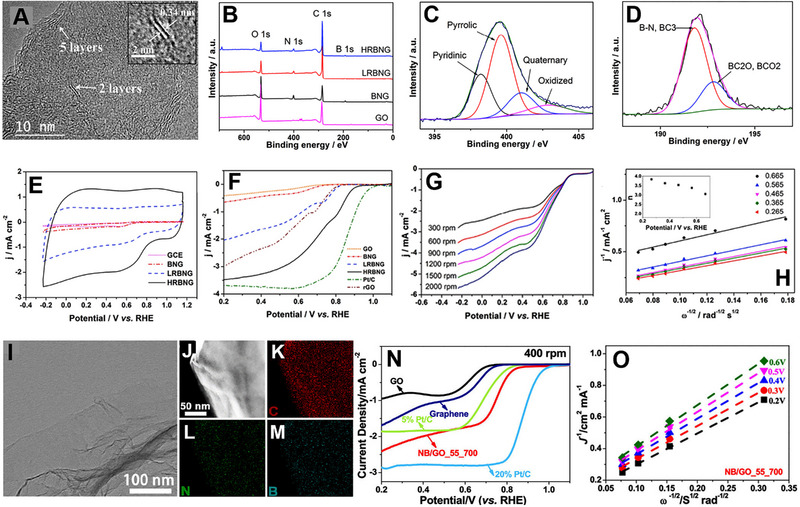
(A) HRTEM image of HRBNG. (B) XPS survey spectra of GO, BNG, LRBNG, and HRBNG. XPS region spectra of (C) N1s and (D) B1s. (E) CVs of GCE, BNG, LRBNG, and RHBNG in O_2_ saturated 0.1 M KOH solutions at a scan rate of 100 mV^‐1^. (F) LSV curves of GO, BNG, LRBNG, HRBNG, Pt/C, and rGO in O_2_ saturated 0.1 m KOH solution at a scan rate of 10 mV s^‐1^ and electrode rotating rate of 1500 rpm. (G) LSV curves of HRBNG at different rotation speeds. (H) K–L plot for the HRBNG at different potentials. Reproduced with permission.^[^ ^115^ ^]^ Copyright 2016, Elsevier. (I) TEM and (J) STEM images of NB/GO_55_700 with corresponding EDX mapping of (K) C, (L) N, and (M) B. (N) LSV curves of graphene, GO, NB/GO_55_700, 5% Pt/C, and 20% Pt/C in O_2_ saturated 0.1 m KOH solution at a scan rate of 5 mV s^‐1^ and electrode rotating rate of 400 rpm. (O) K–L plots of NB/GO_55_700 derived from LSVs recorded at different rotation speeds. Reproduced with permission.^[^ ^116^ ^]^ Copyright 2019, American Chemical Society.

Two or three elements doping on graphene were also studied to enhance the ORR activity compared to single‐element doped graphene. The DFT study suggested that introducing two dopants can modify the electron‐donor characteristics of adjacent carbon atoms, enhancing adsorption of intermediate.^[^ ^114^ ^]^ Shin and co‐workers synthesized a B and N co‐doped graphene (BNG) using a microwave hydrothermal treatment using ammonium biborate as a precursor.^[^ ^115^ ^]^ Later, the BNG was reduced by hydrazine hydrate using hydrothermal treatment to remove the excess oxygen in the graphene layer. A lowly reduced BN co‐doped graphene (LRBNG) and a highly reduced BN co‐doped graphene (HRBNG) were prepared at 393 and 423 K, respectively. Figure 6A shows the HRTEM image of the HRBNG, showing different graphene layers. It is observed that the morphology of the sample does not alter after the reduction process. XPS analysis confirmed the doping of 3.55% B and 4.43% N in the HRBNG. Figure 6B shows the XPS survey spectra of GO, BNG, LRBNG, and HRBNG. A deconvoluted N1s XPS peak showed four different peaks for pyridinic, pyrrolic, quaternary, and oxidized nitrogen functionalities, as seen in Figure 6C. A B1s XPS peak was deconvoluted into two peaks; one for B─N, BC_3,_ and the other for BC_2_O, BCO_2_ components (Figure 6D). CVs of different samples in O_2_ saturated 0.1 m KOH solutions confirmed the positive effect of B and N doping in graphene for ORR (Figure 6E). Further, LSV curves demonstrated the highly active nature of HRBNG with *E*_onset_ of 0.93 V, which is higher than LRBNG (0.84 V) and BNG (0.73 V) (Figure 6F). HRBNG showed a constant *E*_onset_ and the *J*_L_ increases with increase in rotation speed (Figure 6G). K–L plot suggested that HRBNG followed a 4‐electron pathway for the ORR process (Figure 6H).

Sun and co‐workers prepared N and B co‐doped graphene using ultrasonication treatment.^[^ ^116^ ^]^ Ammonia solution and boric acid solution were employed as precursors for N and B doping. The doping amount of N and B can be tuned by ultrasonication and annealing temperatures. The co‐doped sample, which was ultrasonicated at 55°C and annealed at 700°C (termed as NB/GO_55_700), exhibited a 2D layer morphology as confirmed by the TEM analysis (Figure 6I **)**. Scanning TEM (STEM) and EDX analysis suggested that B and N are equally distributed throughout GO (Figure 6J–M). NB/GO_55_700 exhibited higher ORR performance in 0.1 m KOH solution than B/GO_55_700 and N/GO_55_700 (Figure 6N). NB/GO_55_700 showed *E*_onset_ of 0.82 V versus RHE and *J*_L_ of 2.43 mA cm^‐^^2^. Graphitic N, Pyridinic N, and BC_3_ acted as active ORR sites and followed a 4‐electron transfer process generating OH^−^ as the main product (Figure 6O). The synergistic interaction of B and N plays a crucial role in enhancing ORR activity.

**FIGURE 7:**
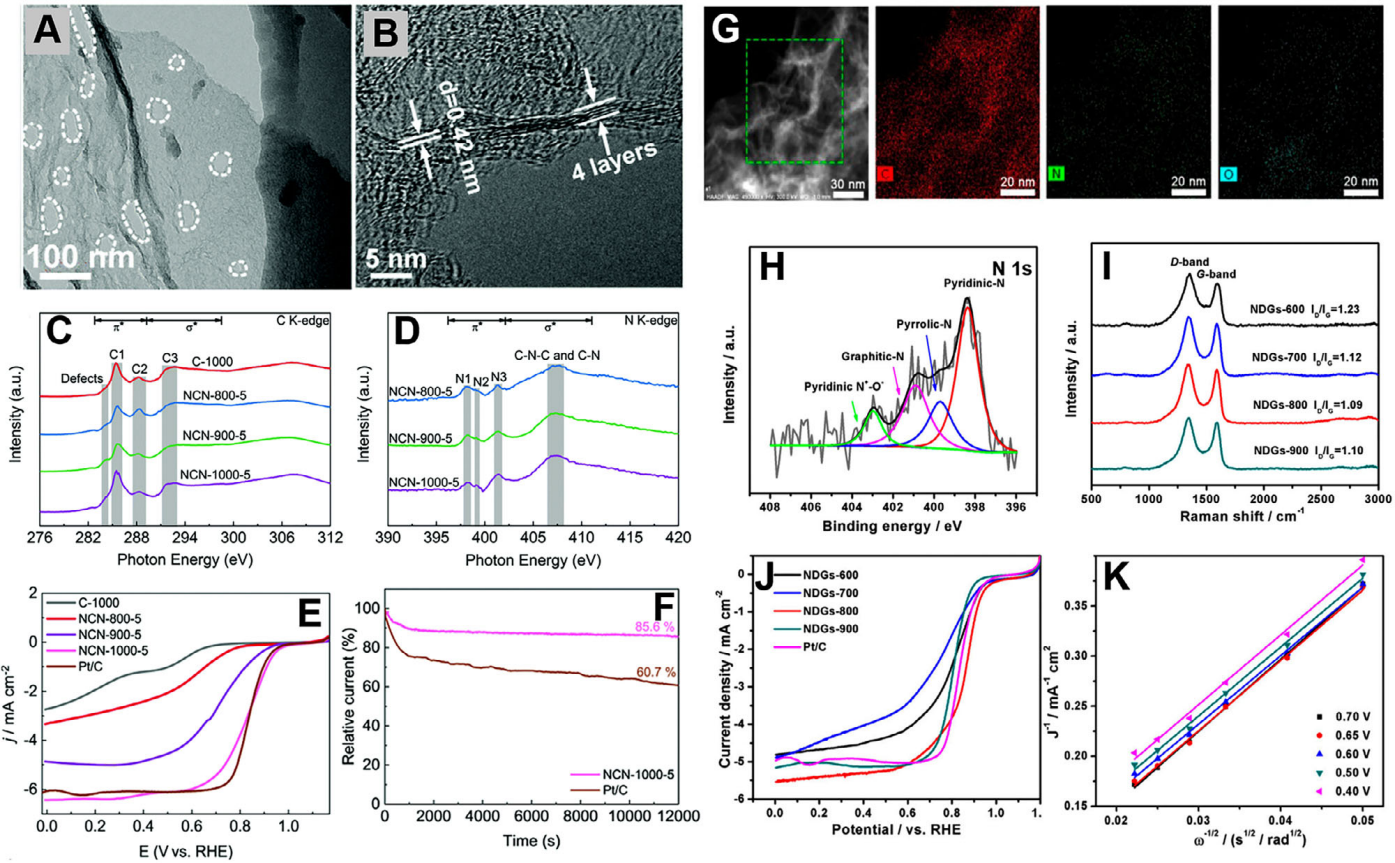
(A) TEM and (B) HRTEM images of NCN‐1000‐5. (C) C K‐edge and (D) N K‐edge NEXAFS spectra of C‐1000, NCN‐800‐5, NCN‐900‐5, and NCN‐1000‐5. (E) LSV of C‐1000, NCN‐800‐5, NCN‐900‐5, NCN‐1000‐5, and Pt/C at a scan rate of 5 mV s^‐1^ and electrode rotating rate of 1600 rpm. (F) Durability tests of NCN‐1000‐5 and Pt/C at 0.67 V (1600 rpm). Reproduced with permission.^[^ ^119^ ^]^ Copyright 2019, Royal Society of Chemistry. (G) STEM and corresponding elemental analysis of NDGs‐800. (H) High‐resolution N 1s XPS spectrum of the NDGs‐800. (I) Raman spectra of NDGs prepared at different temperatures. (J) LSV curves of NDGs prepared at different temperatures and Pt/C catalyst for ORR in 0.1 m KOH at a scan rate of 5 mV s^‐1^. (K) K–L plots of NDGs‐800 at different potential values. Reproduced with permission.^[^ ^120^ ^]^ Copyright 2018, American Chemical Society.

Surface defects can also play a role in increasing the catalytic performance of carbon‐based 2D materials. In the case of graphene, some intrinsic or extrinsic defects can be introduced during synthesis or post‐treatment processes.^[^ ^117^ ^]^ These defects help to adsorb the reactant during the catalytic process. In graphene, defects are always associated with heteroatom doping. However, it is very hard to identify the actual active site, even in the case of a well‐studied N‐doped graphene system.^[^ ^118^ ^]^ Theoretical studies suggested that intrinsic defects in graphene play a huge role in electrocatalytic activity, but it is a big challenge to distinguish the individual defect types and associated roles and characterize experimentally.^[^ ^1^ ^]^ Li and co‐workers introduced defects in N‐doped carbon nanosheets (NCN) using a pyrolysis method.^[^ ^119^ ^]^ The material was synthesized by a simple one‐step carbonization of citric acid and NH_4_Cl. NH_4_Cl acted as a nitrogen source by generating NH_3_ during its thermal decomposition. From TEM analysis, it is observed that the NCN contains mesopores and micropores (Figure 7A). HRTEM further confirmed that the NCN contained four graphitic layers (Figure 7B). The sample prepared at 1000°C possessed a large surface area (1793 m^2^ g^‐1^) and edge defects. Near edge X‐ray absorption fine structure (NEXAFS) study confirmed that in the case of C K‐edge spectra, the defects observed at 284.0 eV were due to the low‐coordination carbon atoms located at the edges of NCNs (Figure 7C). N K‐edge NEXAFS spectra confirmed the presence of graphitic N, pyrrolic N, pyridinic N, C─N, and C─N─C (Figure 7D). DFT calculation suggested that the carbon atoms positioned at the armchair edge and next to the graphitic N dopants act as intrinsic active sites for ORR. The as‐synthesized sample showed *E*_onset_ of 0.95 V versus RHE, *E*_1/2_ of 0.82 V versus RHE, and a high *J*_L_ of 6.43 mA cm^‐^^2^ (Figure 7E). The catalyst also showed a long‐term durability toward ORR (Figure 7F). Ultra‐thin sheet‐like structure, high surface area, and rich in edge defects promoted the ORR activity of NCN. Wang and co‐workers established a defect‐activity relationship for ORR.^[^ ^120^ ^]^ A pyridinic‐N‐doped defective graphene was synthesized via a pyrolysis process. First, a black hydrogel was prepared by hydrothermal treatment of g‐C_3_N_4_ and GO mixture, which was then dried and heated at a temperature range of 600–900°C under N_2_ flow. Thin layers of graphene were obtained along with uniform N distribution confirmed from STEM and elemental mapping (Figure 7G). The sample synthesized at 800°C (NDGs‐800) displayed a maximum percentage of pyridinic N content confirmed from the XPS analysis (Figure 7H). In Raman spectra, the peaks at ≈1350 cm^‐1^ (D band) and ≈1580 cm^‐1^ (G band) correspond to the disorder and the vibration of sp^2^‐bonded carbon atoms, respectively. All the samples showed an *I*_D_/*I*_G_ ratio in a range of 1.09–1.23, confirming highly defective structures (Figure 7I). NDGs‐800 delivered the highest ORR activity among all the catalyst exhibiting *E*_onset_ of 0.98 V versus RHE, *E*_1/2_ of 0.85 V versus RHE, and *J*_L_ of 5.6 mA cm^‐^^2^ (Figure 7J). NDGs‐800 followed a 4‐electron ORR pathway confirmed from K–L plot (Figure 7K). DFT study further confirmed the synergy between pyridinic N‐doped carbon site and a vacancy defect to lower the ORR overpotential of the material.

**FIGURE 8:**
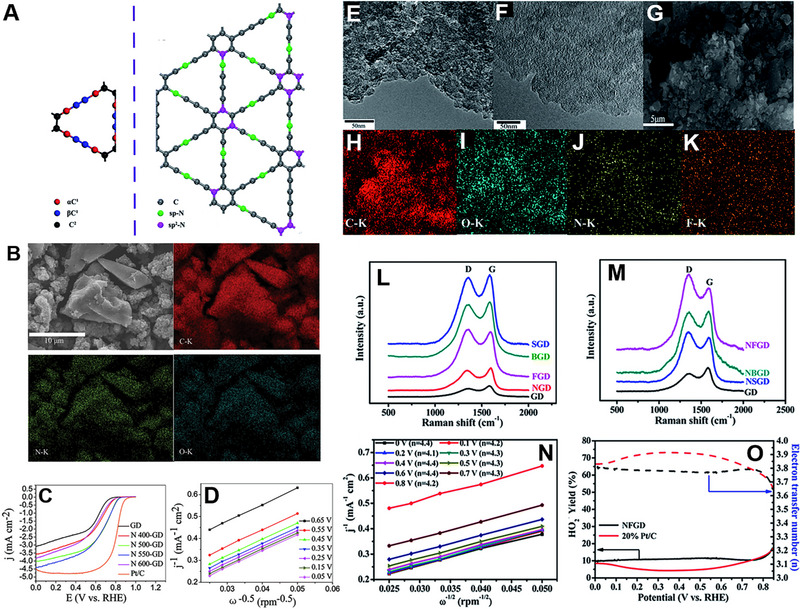
(A) Types of carbons in pristine GY (red: αC^1^; blue: βC^1^; black: C^2^) and position distribution of different N doping in GY. Reproduced with permission.^[^ ^121^ ^]^ Copyright 2020, Royal Society of Chemistry. (B) FESEM and EDS elemental mapping images of the prepared N 550‐GD powder. (C) LSV curves for GD, N‐doped GDs, and Pt/C in O_2_‐saturated 0.1 m KOH solution at a scan rate of 10 mV s^‐1^ with a rotation of 1600 rpm. (D) Koutecky–Levich plots for the N 550‐GD. Reproduced with permission.^[^ ^124^ ^]^ Copyright 2014, Royal Society of Chemistry. TEM images of (E) GD and (F) NFGD. (G) FESEM and (H‐K) EDS elemental mapping images of NFGD. Raman spectra of (L) single elemental doped GD and (M) N, X (X = S, B, F)‐co‐doped GD materials. (N) K–L plots of NFGD calculated at different potentials. (O) HO_2_^−^ yields and electron transfer number of NFGD and 20% Pt/C at various disk electrode potentials obtained from the rotating ring‐disk electrode tests. Reproduced with permission.^[^ ^125^ ^]^ Copyright 2016, Royal Society of Chemistry.

Graphynes (GYs) represent a novel class of carbon allotropes characterized by a 2D planar network structure that combines sp‐ and sp^2^‐hybridized carbon atoms, achieved by linking acetylene bonds with benzene rings. The graph‐*n*‐yne (where *n* = 1, 2, 3, and so on) can be named based on the number of acetylenic chains present between the neighboring benzene rings within a GYs unit.^[^ ^121^ ^]^ The typical structure of GYs is shown in Figure 8A. The primary approach for synthesizing 2D GYs involves bottom‐up methods, like chemical vapor deposition (CVD) and liquid/liquid or gas/liquid processes taking hexaethynyl benzene as a precursor.^[^ ^122^, ^123^ ^]^ It was suggested that certain GY allotropes, characterized by non‐hexagonal symmetry and the presence of two self‐doped non‐equivalent distorted Dirac cones, might display superior electronic properties compared to graphene.^[^ ^1^ ^]^ Furthermore, due to the presence of additional alkyne units between the benzene rings in graphdiyne (GD, *n* value is 2), the network's pore size is enlarged to around 2.5 Å. This enlargement enables adsorption of air into the pores when the sample is introduced in atmosphere.^[^ ^124^ ^]^

Like graphene, the electronic structure of GYs can be tuned via heteroatom doping, resulting in high positive charges on carbon atoms that can enhance the electrocatalytic properties of GYs.^[^ ^125^ ^]^ Zhang and co‐workers reported N‐doped GD as a metal‐free catalyst for the first time.^[^ ^124^ ^]^ To dope the N atom, GD was heated at different temperatures under high‐purity NH_3_ mixed with Ar. Figure 8B shows the SEM and corresponding elemental mapping of the sample prepared at 550°C. XPS analysis suggested that N atom have two different kinds of bonding characteristics, imine 1 N and imine 2 N. Sample prepared at 550°C showed the best performance among all the synthesized materials, having *E*_onset_ of 0.899 V versus RHE (Figure 8C). From the K–L plot, the number of electrons transferred per O_2_ molecule was calculated to be about 3.8 (Figure 8D). The DFT calculation indicated that interaction between O_2_ and imine N‐doped GD is a chemical adsorption, with a significantly stronger binding energy than O_2_ on pyridinic N‐doped GD, which can be categorized as a physical adsorption. The significant elongation of O_2_ molecule bond length from 1.210 Å in pristine O_2_ molecules to 1.376 Å upon adsorption on imine N‐doped GD suggested that parallel diatomic adsorption can efficiently weaken the O─O bonding, facilitating the ORR at the imine N‐doped GD/GC electrodes. Large pore size of the N‐doped GD network also enhances the oxygen adsorption and reduction process, making the material more stable than Pt/C in ORR.

Li and co‐workers doped elements such as B, N, S, and F in GD, and found that N and F co‐doped GD (NFGD) performed similarly to Pt/C in half‐cell and full‐cell.^[^ ^126^ ^]^ The doping was accomplished via a pyrolysis using ammonia, boron oxide, thiourea, and ammonium fluoride as precursors for N, B, S, and F, respectively. TEM, FESEM, and EDS elemental analysis confirmed the uniform doping of N and F in the GD layers (Figure 8E–K). Raman spectra revealed that the *I*_D_/*I*_G_ increases from 0.74 to 0.90 for N doping and further increases to 1.22 for dual atom doping, suggesting the increase of defects in the GD system (Figure 8L,M). NFGD exhibited an average electron transfer number of 4.2 across the reaction, spanning a broad potential range from 0 V to 0.8 V versus RHE, signifying full selectivity toward total oxygen reduction (Figure 8N). HO_2_^−^ yield for NFGD was about 10% in the potential range of 0–0.85 V versus RHE, suggesting the potential of cell application (Figure 8O). DFT calculation suggested that the pure GD does not have catalytic properties, but after doping, the number of active sites increases, promoting the ORR performance. Considering all the examples discussed in this section, it can be concluded that doping heteroatoms and creating defects in the graphene and graphyne greatly enhanced the ORR activity in alkaline media. The enhanced ORR activity parameters of graphene and graphyne catalysts are listed in Table 1.

**TABLE 1:** Summary of N‐doped graphene catalysts for the ORR.

Catalyst	Synthetic method	Electrolyte	*E*_onset_ (vs RHE)	*E*_1/2_ (vs RHE)	Electron transfer (*n*)	Tafel slope (mV dec^‐1^)	*J*_L_ (mA cm^‐1^)	Ref.
NrGO800	Thermal annealing	0.1 m NaOH	0.88 V	0.76 V	3.66	65.4		[99]
NrGO‐900	Pyrolysis	0.1 m KOH	0.99 V	0.84 V	4	–		[100]
NrGO	Thermal annealing	0.1 m NaOH	1.03 V	0.84 V	3.5	–		[103]
NGS4‐900	Pyrolysis	0.1 m KOH	0.984 V	0.859 V	3.9	72	5.98	[102]
PCNs	Thermal annealing	0.1 m KOH	−0.02 V (vs Ag/AgCl)	–	3.49–3.67	–	4.6	[127]
N‐doped graphene	Hydrothermal and microwave treatments	0.1 m KOH	−0.056 V (vs Ag/AgCl)	–	≈4	–	3.91	[128]
1‐NGF‐9	Thermal annealing	0.1 m KOH	0.89 V	–	≈4	–	5.30	[129]
10N‐G‐800	Pyrolysis	0.1 m KOH	0.930 V	–	3.99	60	5.13	[130]
N‐G‐1000	Pyrolysis	0.1 m KOH	0.982 V	0.862 V	3.92	72	5.48	[131]
B‐rGO	Hydrothermal	0.1 m KOH	0.833 V	0.671 V	3.7	76	4.1	[112]
PN‐BG	Hydrothermal treatment and electrochemical deposition	0.1 m KOH	–	–	3.8–4.0	–	–	[113]
S‐doped graphene nanosheet	Electrochemical exfoliation	0.1 m NaOH	0.810 V	–	3.93	−50.8	–	[132]
F‐doped graphene	Thermal treatment	0.1 m KOH	−0.28 V (vs Ag/AgCl)	–	3.9–4.05	55		[133]
N, S co‐doped Graphene	Three‐step pyrolysis	0.1 m KOH	1.01 V	0.870 V	3.975	–	5.99	[134]
B&N‐rGO	Pyrolysis	0.1 m KOH	0.850 V	–	3.5	61.8	–	[135]
B,N‐Graphene	Two‐step pyrolysis	0.1 m KOH	0.06 V (vs Ag/AgCl)	–	–	–	13.87	[114]
HRBNG	Microwave hydrothermal	0.1 m KOH	0.93 V	–	3.84	–	–	[115]
NB/GO_55_700	Ultrasonication and thermal annealing	0.1 m KOH	0.82 V	–	3.98	43	2.43	[116]
NBC‐1000	Thermal annealing	0.1 m KOH	0.97 V	0.84 V	3.93	–	5.4	[136]
P‐N‐Gr	Two step thermal annealing	0.1 m KOH	1.005 V	0.82 V	3.95	67	5.98	[137]
NP‐PrGO‐1100	Pyrolysis	0.1 m KOH	0.913 V	0.819 V	4	78	5.48	[138]
NCN‐1000‐5	Pyrolysis	0.1 m KOH	0.95 V	0.82 V	3.92	86	6.43	[119]
NDGs‐800	Pyrolysis	0.1 m KOH	0.98 V	0.85 V	≈4	81	5.6	[120]
N‐doped carbon nanoribbon	Pyrolysis	0.1 m KOH	0.99 V	0.870 V	3.9	78	5.8	[139]
N and S co‐doped carbon	Wet chemical	50 mm PBS	0.769 V	0.613 V	3.76–4.02	–	4.773	[140]
N and P co‐doped porous carbon	Pyrolysis	0.1 m KOH	0.98 V	0.870 V	3.93	–	6.24	[141]
N‐doped carbon/graphene	Pyrolysis	0.1 m KOH	0.922 V	0.524 V	–	–	1.85	[142]
N‐doped graphitic porous carbon	Carbonization	0.1 m KOH	−0.04 V (vs Ag/AgCl)	−0.18 V (vs Ag/AgCl)	3.92	–	5.7	[143]
S, N dual‐doped graphene‐like carbon nanosheets	Pyrolysis	0.1 m KOH	0.950 V	0.830 V	3.6–3.9	–	4.86	[144]
N‐doped graphdiyne	Pyrolysis	0.1 m KOH	0.899 V	–	3.8	–	−4.5	[124]
N, F co‐doped graphdiyne	Pyrolysis	0.1 m KOH	1.0 V	–	4.2	–	–	[125]
N‐doped few‐layer graphdiyne	Pyrolysis	0.1 m KOH	–	0.87 V	3.9	60	–	[145]
N‐doped porous graphdiyne	Pyrolysis	0.1 m KOH	0.98 V	0.83 V	3.5	74	5.1	[146]
N, P‐co‐doped graphdiyne	Thermal annealing	0.1 m KOH	–	–	3.62–3.68	58	–	[147]

### 3.2 Graphitic carbon nitride (g‐C_3_N_4_)

**FIGURE 9:**
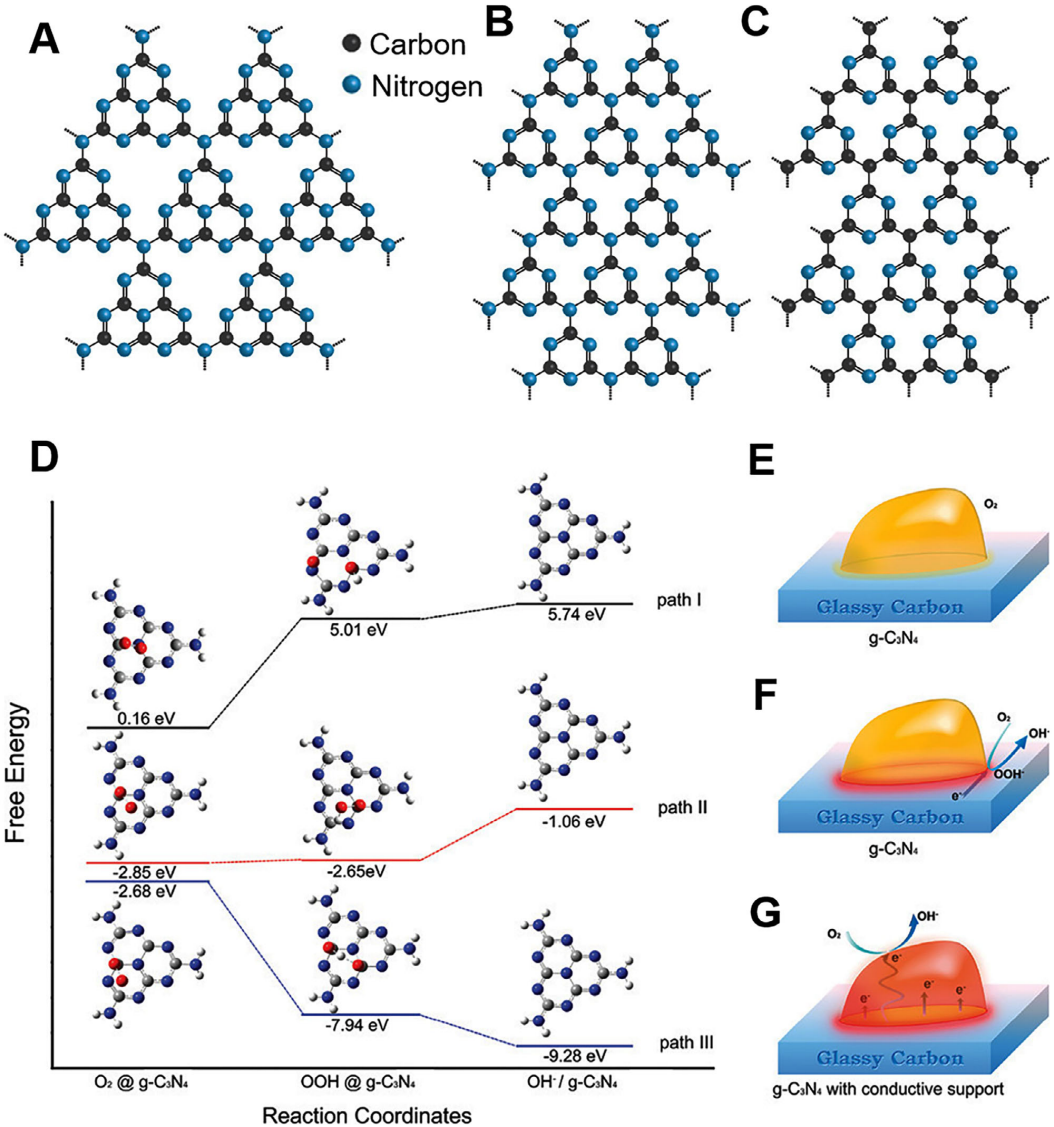
Different structural motifs of (A) polyheptazine g‐C_3_N_4_, (B) polytriazine g‐C_3_N_4_, (C) polytriazine g‐C_4_N_3_. Reproduced with permission.^[^ ^155^ ^]^ Copyright 2018, American Chemical Society. (D) Free energy plots of ORR and optimized configurations of adsorbed species on the g‐C_3_N_4_ surface with zero, two, and four electron participation demonstrated as paths I, II, and III. Gray, blue, red, and white small spheres representing C, N, O, and H. Schematics of ORR pathways on (E) pristine g‐C_3_N_4_ without electron participation, (F) pristine g‐C_3_N_4_ with 2e^–^ participation, and (G) g‐C_3_N_4_ and conductive support composite with 4e^–^ participation. Reproduced with permission.^[^ ^150^ ^]^ Copyright 2011, American Chemical Society.

The g‐C_3_N_4_ is widely acknowledged for its exceptional stability as a stable allotrope, making it a remarkable 2D material with significant potential in energy‐related applications. The generation of a 2D framework with N heteroatoms substituted into graphite occurs through the sp^2^ hybridization of N and C atoms.^[^ ^148^ ^]^ Previous studies have identified tri‐s‐triazine as the fundamental unit of the g‐C_3_N_4_ network. Different types of graphene nitride structures are shown in Figure 9A–C. In theory, g‐C_3_N_4_ showed an abundance of pyridine‐like N atoms within the heptazine heteroring, offering an ample supply of electron lone pairs, making them active sites for ORR. However, the limited conductivity posed a significant obstacle to its practical application in electrocatalysis.^[^ ^149^ ^]^ Free energy calculation indicated that the restricted electron transfer capacity of g‐C_3_N_4_ results in a build‐up of OOH^−^ intermediates on the catalyst surface, primarily due to an ineffective 2e^−^ reduction process (Figure 9D).^[^ ^150^ ^]^ In the case of path I, oxygen reduction cannot occur naturally on the g‐C_3_N_4_ surface in the absence of electron involvement because of the presence of two high energy barriers in the free energy plot corresponding to intermediate and final products (Figure 9E). Upon the introduction of electrons (via path II), the free energy of the intermediate OOH@g‐C_3_N_4_ was reduced to a level comparable to that of the initial state of O_2_@g‐C_3_N_4_. This suggests that the initial 2e^−^ reaction can proceed unhampered. However, an evident barrier remains at the last state of OH^−^/g‐C_3_N_4_, preventing the progression of the second 2e^−^ reaction (Figure 9F). Incorporating a conducting material with g‐C_3_N_4_ increased the electron transfer efficiency and facilitates a 4e^−^ transfer process (Figure 9G). Various types of carbon materials have been incorporated to increase the electron transfer efficiency and thus the ORR efficiency of g‐C_3_N_4_.^[^ ^151^, ^152^, ^153^, ^154^ ^]^

**FIGURE 10:**
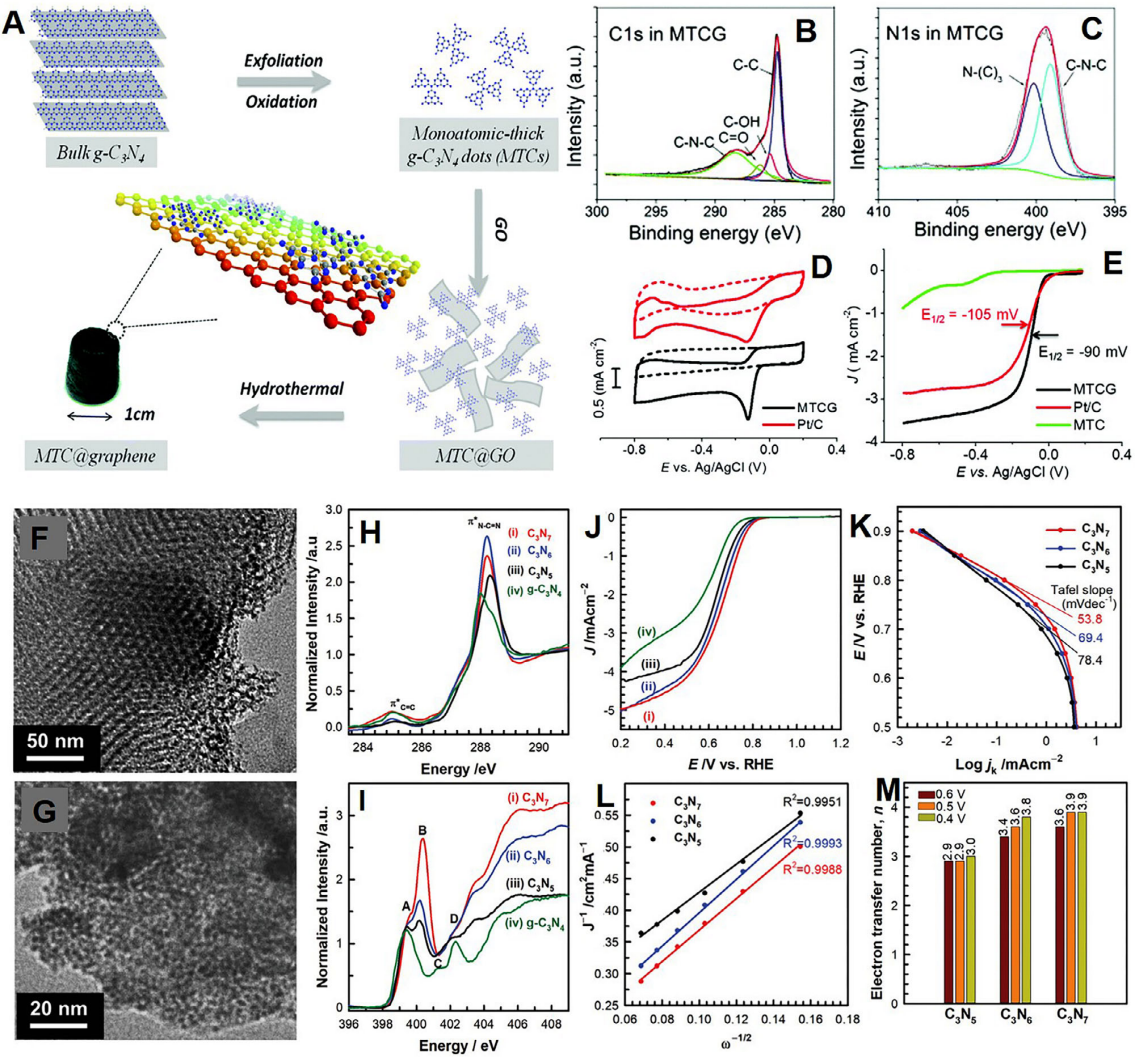
(A) Schematic of monoatomic‐thick g‐C_3_N_4_ dots@graphene (MTCG) synthesis procedure. XPS spectra of MTCG (B) C1s and (C) N1s. (D) CVs of MTCG and Pt/C catalysts at a scan rate of 10 mV s^‐1^ in O_2_‐saturated (solid lines) or N_2_‐saturated (dashed lines) 0.1 m KOH solution. (E) RRDE polarization curves of MTCG and Pt/C catalysts with a scan rate of 10 mV s^‐1^ at 1200 rpm in O_2_‐saturated 0.1 m KOH solution. Reproduced with permission.^[^ ^156^ ^]^ Copyright 2015, Royal Society of Chemistry. HRTEM images of (F) C_3_N_7_ and (G) C_3_N_6_. (H) C K‐ and (I) N K‐edge NEXAFS spectra of C_3_N_7_ and C_3_N_6_ with reference materials. (J) LSV of (i) C_3_N_7_, (ii) C_3_N_6_, (iii) C_3_N_5_, and (iv) g‐C_3_N_4_ at 1600 rpm. (K) Tafel plots of C_3_N_7_, C_3_N_6_, and C_3_N_5_ at 1600 rpm. (L) K–L plots at 0.6 V versus RHE, and (M) electron transfer number of the C_3_N_7_, C_3_N_6,_ and C_3_N_5_ calculated by K–L plots at 0.6, 0.5, and 0.4 V versus RHE. Reproduced with permission.^[^ ^157^ ^]^ Copyright 2021, Wiley‐VCH.

Qu and co‐workers synthesized a heterostructure by combining the basal plane of the graphene sheet with monolayer g‐C_3_N_4_ dots (MTCG).^[^ ^156^ ^]^ Figure 10A shows the schematic of the synthesis process. The g‐C_3_N_4_ was oxidatively exfoliated by a modified Hummers method and was then mixed with GO. The overall solution was hydrothermally treated to obtain MTCG. STEM elemental mappings showed that the C and N elements are uniformly distributed all over the material, confirming the even distribution of g‐C_3_N_4_ across the graphene surface. XPS analysis further confirmed the formation of MTCG (Figure 10B,C). In the case of C1 spectra, the peak at 288 eV was assigned to sp^2^ carbon atoms within the triazine rings, which are connected to nitrogen atoms within the aromatic structure group or the ‐NH_2_ group arising from g‐C_3_N_4_. On the other hand, the peak at 284.6 eV was designated to graphitic carbon originating from graphene. The deconvoluted N1s spectra show two individual peaks, one for s‐triazine rings (C─N─C, 398.8 eV) and another for bridging N atoms in N─(C)_3_ (400.1 eV). The ORR performance of MTCG was carried out in 0.1 m KOH solution, and results were compared with commercial Pt/C. MTCG showed an oxygen reduction peak at −0.13 versus Ag/AgCl, close to the Pt/ C value (Figure 10D). MTCG showed *E*_onset_ of −0.02 V versus Ag/AgCl and *E*_1/2_ of −0.09 V versus Ag/AgCl, both lower than the commercial Pt/C (Figure 10E). Both MTCG and Pt/C showed a two‐stage feature in overall reaction kinetics. In the lower potential, a small slope was observed, which is controlled by the surface reaction rate. However, a steeper curve was observed in the higher potential because the ORR is more relied on oxygen diffusion. Tafel slopes of MTCG are 59 and 240 mV dec^‐1^, which are close to 65 and 210 mV dec^‐1^ values of Pt/C. Monolayer g‐C_3_N_4_ dots (MTC) exhibited a reduced onset potential, half‐wave potential, and limiting current density when compared to MTCG, confirming the beneficial impact of the 2D graphene layer structure on ORR.

Synthesis of carbon nitride with an N:C atomic ratio > 2 is challenging due to substituting C─N bonds with thermodynamic less stable N─N bonds. A higher ratio is believed to offer superior basicity and unique electronic properties. Vinu and co‐workers synthesized C_3_N_6_ and C_3_N_7_ using a low‐temperature pyrolysis method.^[^ ^157^ ^]^ 5‐amino‐1H‐tetrazole and KIT‐6 were mixed together and heated at 160°C for 6 h. The resulting product was further calcined at 250 and 300°C under N_2_ atmosphere for 4 h to form mesoporous C_3_N_7_ and C_3_N_6_, respectively. TEM analysis suggested that the materials have highly ordered 2D porous structures (Figure 10F,G). The N─N bonds were stabilized by forming tetrazine and/or triazole moieties, confirmed by near‐edge X‐ray absorption fine structure (NEXAFS) analysis (Figure 10H,I). C_3_N_7_ and C_3_N_6_ showed better ORR performance and lower Tafel slopes than C_3_N_5_ and g‐C_3_N_4_ (Figure 10J,K). The K‐L plots of the materials implied that the material with higher N content showed a higher *n* number, suggesting an effective reduction of O_2_ to 4OH^−^ (Figure 10L,M). According to DFT calculation, C_3_N_7_ contained a higher amount of cyclic N─N bonds, which effectively absorb O_2_ and OH species, making it more ORR active. The enhanced ORR activity parameters of g‐C_3_N_4_ catalysts are listed in Table 2. Therefore, a heterostructure of g‐C_3_N_4_ is much more electrocatalytically active than pure g‐C_3_N_4_ in ORR.

**TABLE 2:** Summary of g‐C_3_N_4_ for the ORR.

Catalyst	Synthetic method	Electrolyte	*E*_onset_ (vs RHE)	*E*_1/2_ (vs RHE)	Electron transfer (*n*)	Tafel slope (mV dec^‐1^)	*J*_L_ (mA cm^‐1^)	Ref.
Graphitic‐C_3_N_4_@Carbon	Template method	0.1 m KOH	−0.10 V versus Ag/AgCl	–	3.8	−113	11.3	[150]
C_3_N_7_	Pyrolysis	0.1 m KOH	0.81 V	–	3.9	53.8	8.2	[157]
g‐C_3_N_4_ dots@graphene	Hydrothermal	0.1 m KOH	−0.02 V versus Ag/AgCl	0.09 V versus Ag/AgCl	3.72	59, 240	–	[156]
g‐C_3_N_4_@N‐G	Ball milling	0.1 m KOH	−0.02 V versus SCE	−0.22 V versus SCE	4	–	–	[158]
Graphene platelet decorated carbon nitride	Pyrolysis	0.1 m KOH	0.87 V	–	3.8	63	5.1	[159]
g‐C_3_N_4_@rGO	Semi‐closed pyrolysis	0.1 m KOH	−0.248 V versus Ag/AgCl	−0.281 V versus Ag/AgCl	3.21	–	3.36	[160]
g‐C_3_N_4_ QD/g‐C_3_N_4_ sheet/rGO	Ultrasonication	0.1 m KOH	−0.075 V versus Ag/AgCl	−0.210 V versus Ag/AgCl	3.9–4.2	–	−0.491	[161]
s‐g‐C_3_N_4_@GQDs	Hydrothermal	0.1 m KOH	−0.07 V versus Ag/AgCl	–	3.5	–	–	[162]
S‐doped C_3_N_4_‐mesoporous carbon	Polycondensation	0.1 m KOH	−0.11 V versus Ag/AgCl	–	4	–	30.9	[163]

## 4 NON‐CARBON‐BASED 2D ELECTROCATALYSTS FOR THE ORR

As seen in the previous section, carbon‐based materials, mainly graphene, have been extensively studied for the ORR. In this section, the advancements of other metal‐free 2D nanomaterials, such as hexagonal boron nitride (h‐BN), boron carbon nitride (BCN), and black phosphorus (BP) for the ORR are discussed.

### 4.1 h‐BN

Recently, BN has gained significant attention for green energy conversion due to its remarkable optoelectrical properties, thermal stability, mechanical durability, and chemical inertness. Four types of BN have been reported in the literature, including amorphous BN (a‐BN), h‐BN, cubic BN (c‐BN), and wurtzite BN (w‐BN).^[^ ^164^ ^]^ h‐BN exhibits a layered structure similar to graphene, composed of sp^2^ hybridized B─N bonds that are highly polarized and possess a strong covalent character within the plane. However, unlike graphene, h‐BN displays significant polarization due to the electronegativity of N atoms, leading to the potential for anisotropic properties. Due to the huge gap in electronegativity between B and N, h‐BN behaves as an insulator, and the band gap depends on the thickness of the material. Although h‐BN has been used in optical and electrical devices as a dielectric substrate, h‐BN remains inert toward electrocatalysis.^[^ ^1^, ^164^, ^165^ ^]^ Utilization of pristine h‐BN for the ORR in fuel cells is impractical due to its inherent limitations in electronic conductivity and its tendency to form H_2_O_2_ through a two‐electron process.^[^ ^166^ ^]^ To activate the h‐BN, various methods have been adopted, such as physical methods and chemical methods. There are reports of adding non‐metal nanostructures with h‐BN to increase its electrocatalytic activity. Doping carbon into the h‐BN matrix or forming a heterostructure between graphene and h‐BN might resolve this issue.^[^ ^167^, ^168^ ^]^

**FIGURE 11:**
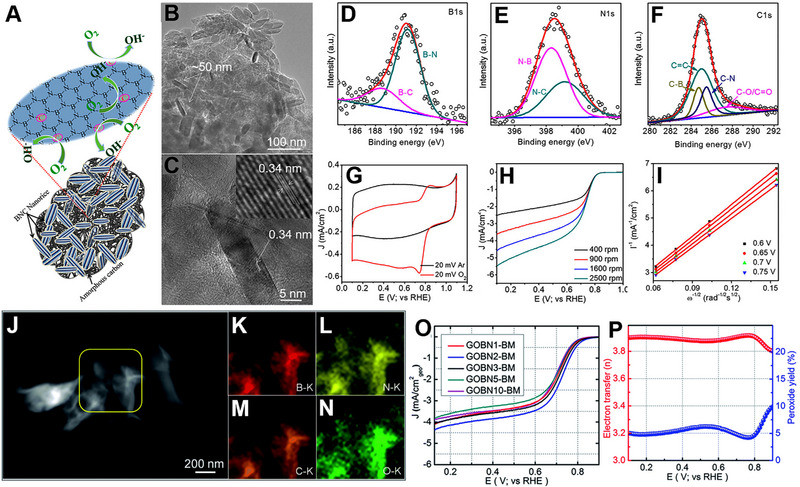
(A) Schematic illustration for ORR of carbon‐doped h‐BN (BNC) catalyst. (B) TEM and (C) HRTEM images of BNC catalyst. Deconvoluted XPS spectra of (D) B1s, (E) N1s, and (F) C1s. (G) CVs of BNC catalyst, recorded in Ar‐ and O_2_‐saturated 0.1 m KOH at a scan rate of 20 mV s^‐1^. (H) ORR polarization curves of BNC catalyst under O_2_‐saturated 0.1 m KOH recorded at various rotation speeds (I) K‐L plot of BNC catalyst at different potentials. Reproduced with permission.^[^ ^167^ ^]^ Copyright 2018, American Chemical Society. (J) HAADF‐STEM image of the GOBN2–BM catalyst and (K–N) corresponding elemental mapping of B, N, C, and O. (O) Comparison of the ORR LSV curves of electrocatalysts with varied h‐BN amount in O_2_‐saturated 0.1 m KOH at 1600 rpm with a scan rate of 10 mV s^‐1^. (P) Extent of peroxide yield and number of electrons transferred (*n*) of the GOBN2–BM catalyst in the potential range of 0.1 to 0.9 V versus RHE. Reproduced with permission.^[^ ^169^ ^]^ Copyright 2018, Royal Society of Chemistry.

Utilizing a CVD method, Kakade and co‐workers prepared a one‐step carbon‐doped h‐BN (BNC).^[^ ^167^ ^]^ Figure 11A represents the schematic of BNC for ORR. Boric acid and hexamethylenetetramine were mixed in a solid‐state condition and then heated for 2 h under an N_2_ atmosphere. TEM analysis of the sample prepared at 850°C confirmed that the rice‐shaped BNC nanocrystals were impregnated into the carbon matrix (Figure 11B,C). XPS region spectra of B1s and N1s suggested the formation of B─N bond and a small amount of B─C and N─C bonds (Figure 11D,E). C1s XPS spectrum also indicated the formation of C─B, C─O, and C─N bond formation along with the C─C bond (Figure 11F). A strong cathodic peak at 0.76 V versus RHE in the presence of O_2_ was observed, but absence in N_2_, confirms the potential of BCN for ORR (Figure 11G). An *E*_onset_ of 0.83 V versus RHE and *J*_L_ of 4.6 mA cm^‐^^2^ were observed at 1600 rpm (Figure 11H). The number of electrons per O_2_ molecule was ≈3.6, calculated from K–L plots at different potentials (Figure 11I). They further confirmed that the carbon coating on BNC increases the active sites, making it more ORR active compared to pure h‐BN. A similar kind of ORR enhancement was observed when rGO was added to h‐BN to make a composite.^[^ ^168^ ^]^ Multiple contacts between rGO and h‐BN act as an oxygen adsorption site and enhance the performance. In another work, Kakade and co‐workers synthesized a heterostructure combining rGO and h‐BN using a single‐step hydrothermal method.^[^ ^169^ ^]^ Initially, GO was mechanically activated via a ball milling process and later mixed with h‐BN heated at 180°C for 6 h in an autoclave. HAADF‐STEM study suggested the generation of a homogeneous composite (Figure 11J–N). The amount of h‐BN was varied to obtain different compositions of heterostructures. Out of all the samples, the heterostructure containing 2% h‐BN (GOBN2‐BM) showed maximum performance toward ORR (Figure 11O). The average number of electrons per O_2_ molecule was 3.89, with a peroxide yield below 10% (Figure 11P). The addition of h‐BN decreased the crystallite size of the GO, subsequently enhancing the active surface area of the material, resulting in higher electrocatalytic activity. The enhanced ORR activity parameters of h‐BN catalysts are listed in Table 3. From the above discussion and results, it is clearly visible that the ORR performance of h‐BN is enhanced either by doping or by forming a heterostructure.

**TABLE 3:** Summary of h‐BN for the ORR.

Catalyst	Synthetic method	Electrolyte	*E*_onset_ (vs RHE)	*E*_1/2_ (vs RHE)	Electron transfer (*n*)	Tafel slope (mV dec‐1)	*J*_L_ (mA cm^‐1^)	Ref.
C‐doped h‐BN	CVD	0.1 m KOH	0.83 V	–	3.6	–	1.58	[167]
rGO/h‐BN	Hydrothermal and annealing	0.1 m KOH	0.798 V	0.64 V	3.7	–	–	[168]
rGO/h‐BN	Hydrothermal	0.1 m KOH	0.98 V	0.74 V	3.89	102	4.4	[169]
CNT/h‐BN	Hydrothermal and annealing	0.1 m KOH	0.86 V	0.72 V	3.9	–	5.78	[170]
BN‐Graphene	EDC Coupling	0.1 m KOH	–	–	3.8	–	−4.6	[171]
Graphene‐hBN	ball milling	0.1 m KOH	0.79 V	–	2.23	–	–	[172]

### 4.2 BCN

**FIGURE 12:**
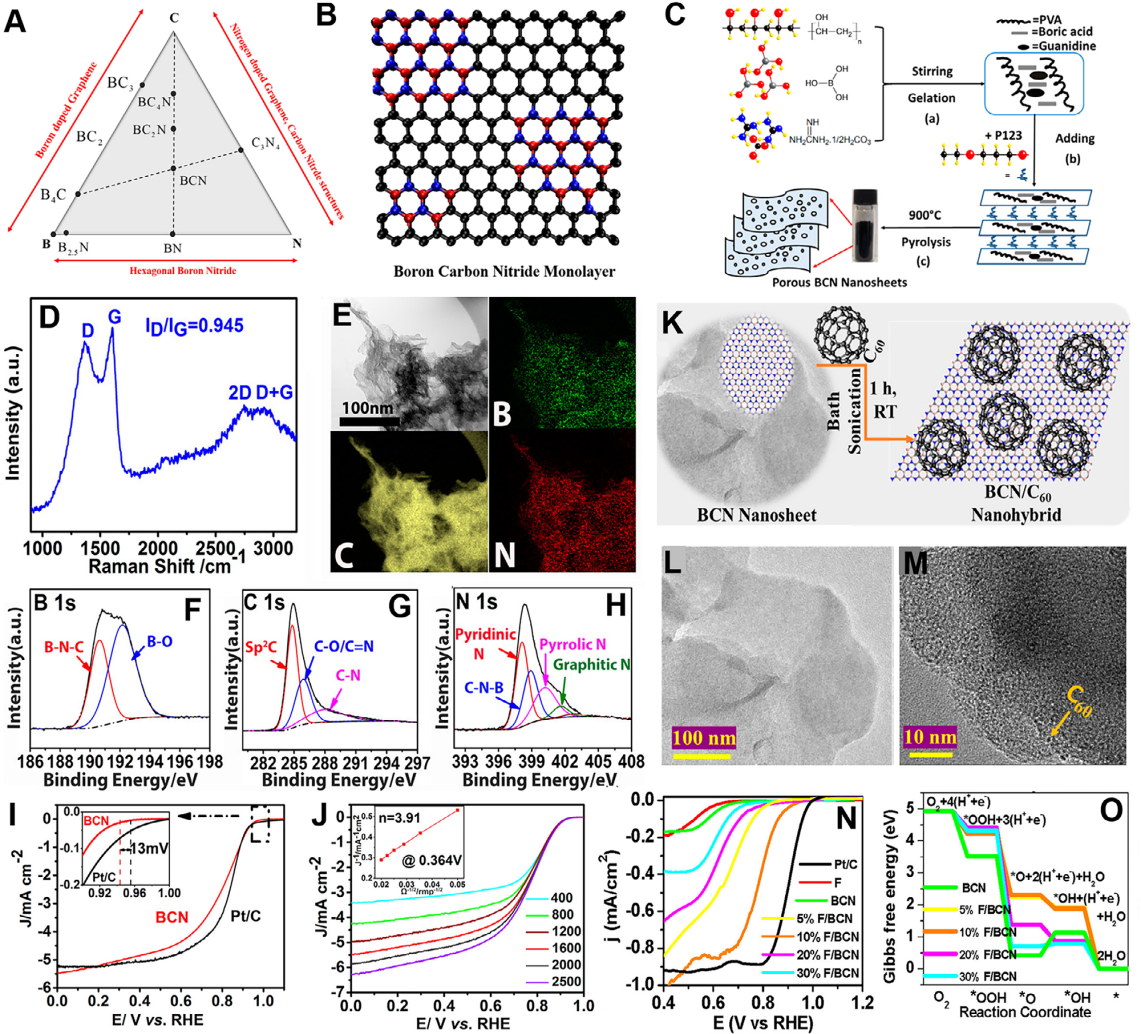
(A) Phase diagram of B–C–N showing possible stable intermediate phases. (B) Schematic illustration of boron carbon nitride crystal structure. Reproduced with permission.^[^ ^176^ ^]^ Copyright 2020, The Electrochemical Society. (C) Schematic of porous BCN nanosheet synthesis. (D) Raman spectra of porous BCN nanosheet. (E) TEM and corresponding elemental mapping of B, C, and N elements. XPS region spectra of (F) B1s, (G) C1s, and (H) N1s. (I) LSVs of BCN and Pt/C in 0.1 m KOH at a rotation rate of 1600 rpm and a scan rate of 5 mV s^‐1^. The inset showing the higher magnified LSV curves between 0.9 and 1.0 V versus RHE. (J) LSVs of BCN at different rotation rates. The inset showing the corresponding K–L plot. Reproduced with permission.^[^ ^175^ ^]^ Copyright 2017 American Chemical Society. (K) Schematic representation of the synthesis of fullerene/BCN nanohybrids. TEM images of (L) BCN nanosheets and (M) 10% Fullerene/BCN nanohybrids. (N) LSV curves of F, BCN, 5% fullerene/BCN, 10% fullerene/BCN, 20% fullerene/BCN, 30% fullerene/BCN, and Pt/C for ORR at 2 mV s^‐1^ in 0.5 m NaOH. (O) Gibbs free energy diagram showing the differences of each elementary ORR catalytic step for both BCN and fullerene/BCN nanohybrids. Reproduced with permission.^[^ ^177^ ^]^ Copyright 2021 American Chemical Society.

Recently, BCN has been getting much attention due to its diverse composition. BCN has a bandgap of 1.18 eV, much lower than h‐BN (5 eV), which is useful for optoelectronic applications.^[^ ^173^ ^]^ BCN is a ternary 2D compound with the advantages of graphene, BN nanosheets, and CN analogs.^[^ ^174^ ^]^ Figure 12A,B shows phase diagram and crystal structure of BCN monolayer, respectively. Like graphene and h‐BN, BCN is also explored for electrocatalytic applications. Lei and co‐workers prepared porous BCN nanosheets using a polymer sol‐gel method.^[^ ^175^ ^]^ Figure 12C shows the schematic of the synthesis process. Initially, a polymeric gel was formed by hydroxyl and amino group cross‐linking between polyvinyl alcohol (PVA), boric acid, and guanidine carbonate salt. The polymeric precursor was subjected to additional curing after the introduction of P123. Following heat treatment at 900°C in an N_2_ atmosphere, the architecture of the precursor gel was gradually transformed into a 2D porous layered structure, resulting in the bonding of B, N, and C atoms. From XRD and FT‐IR spectra, it is noticed that the peak position of BCN is different from the graphene and h‐BN. Raman analysis showed that BCN exhibits D and G bands at ≈1360 and 1600 cm^‐1^ (Figure 12D). Graphene generally shows a G band at 1580 cm^‐1^, which confirms the structural distortion of BCN due to the existence of different bond lengths of B─N and C─N. TEM and EDX showed that all the B, N, and C atoms are uniformly distributed in the 2D BCN nanosheets (Figure 12E). XPS results confirmed the presence of B─O and B─N─C bonds in B1s region spectra, whereas sp^2^ C═C bonds, C═N bonds, C─O/C─N bonds were observed in C1s region spectra (Figure 12F,G). N1s XPS region spectrum showed four peaks corresponding to pyrrolic N, pyridinic N, quaternary N, and C─N─B (Figure 12H). A higher amount of pyridinic N presence in the material is believed to be an active site for ORR. BCN showed *E*_onset_ of 0.940 V versus RHE, which is 13 mV less than commercial Pt/C (Figure 12I). K–L plots suggested that the ORR process followed a four‐electron pathway (Figure 12J). In RRDE measurement, the calculated H_2_O_2_ yield is below 6%, confirming the 4e^−^ transfer process in the alkaline ORR process. The BCN material also showed excellent methanol tolerance and high durability compared to commercial Pt/C. It is believed that the abundant B─N─C bonding in the material is readily available to OH adsorption and O protonation in the graphitic carbon edge area, resulting in higher ORR activity.

Noveron and co‐workers developed a BCN‐based heterostructure catalysis for ORR.^[^ ^177^ ^]^ BCN was prepared through carbonization of the mixture of urea, boric acid, and PEG‐2000 precursors. Later, they added varied amounts of fullerene to BCN to form the heterostructure in an ultrasonication method (Figure 12K). Pure BCN exhibited a smooth 2D structure (Figure 12L), whereas fullerene was uniformly dispersed in the BCN sheet in the case of fullerene/BCN composite (Figure 12M). Higher amount of pyridinic N and C─N─B were detected in the 10% fullerene/BCN composite, implying a higher number of active ORR sites. 10% fullerene/BCN composite showed *E*_onset_ of 0.920 V versus RHE in 0.5 m NaOH solution, which is higher compared to other composites and pristine BCN (Figure 12N). DFT calculation predicted that the *O → *OH step is the rate‐limiting step for all the fullerene/BCN composite. The composite containing 10% fullerene/BCN displayed the least rate‐limiting potential (0.81 eV), leading to a reduction in the energy barrier for the rate‐determining catalytic step, and subsequently, an enhancement in the reaction rate (Figure 12O). The enhanced ORR activity parameters of BCN catalysts are listed in Table 4. Although pure BCN or heterostructure of BCN showed promising results, further study is needed to explore its full potential.

**TABLE 4:** Summary of BCN for the oxygen reduction reaction.

Catalyst	Synthetic method	Electrolyte	Onset potential (vs RHE)	*E*_1/2_ (vs RHE)	Electron transfer (*n*)	Tafel slope (mV dec^‐1^)	*J*_L_ (mA cm^‐1^)	Ref.
BCN	Pyrolysis	0.1 m KOH	0.940 V	0.820 V	3.91	–	–	[175]
BCN/C_60_	Pyrolysis	0.5 m NaOH	0.920 V	0.790 V	3.85	87	–	[177]
BCN/C	Pyrolysis and solvothermal	0.1 m KOH	1.01 V	0.860 V	≈4	72.4	–	[178]
NBC nanosheets	Pyrolysis	0.1 m KOH	0.87 V	–	≈4	79	4.18	[179]

### 4.3 Black phosphorus

**FIGURE 13:**
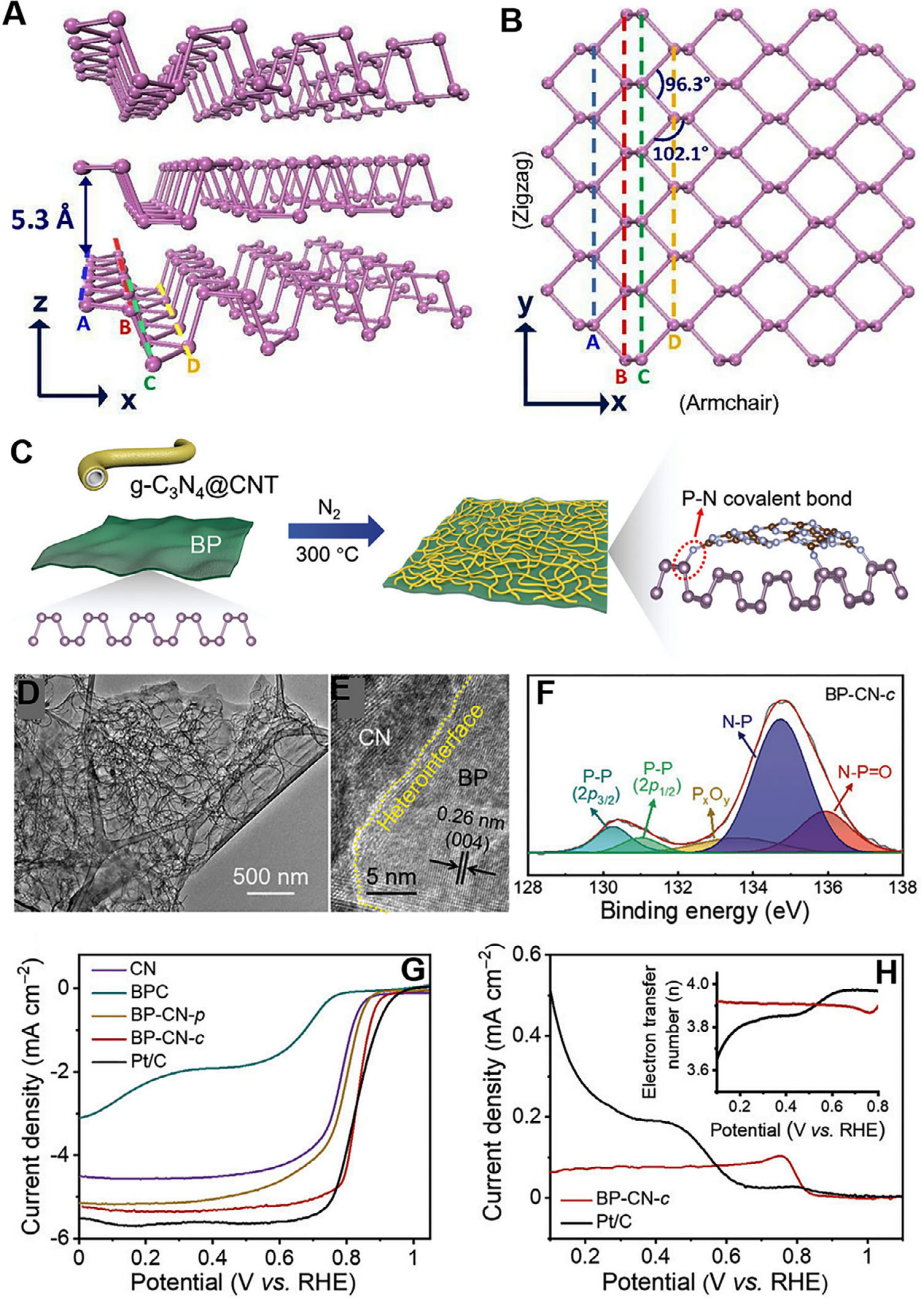
Crystal structure of BP (A) side view and (B) top view. Reproduced with permission.^[^ ^186^ ^]^ Copyright 2015, National Academy of Sciences. (C) Schematic representation of BP‐CN‐c synthesis. (D, E) TEM and HRETM images of BP‐CN‐c (F) P 2p region XPS spectrum (G) ORR polarization curves of CN, BPC, BP‐CN‐p, BP‐CN‐c, and Pt/C at 5 mV s^‐1^ with a rotating speed of 1600 rpm. (H) Ring current densities and the calculated electron transfer numbers (inset) for BP‐CN‐c and Pt/C. Reproduced with permission.^[^ ^189^ ^]^ Copyright 2021, Wiley‐VCH.

The BP is a layered semiconductor material that is artificially synthesized, belonging to the class of monoatomic 2D van der Waals materials.^[^ ^180^, ^181^ ^]^ The individual layers of BP, known as phosphorene, exhibit a puckered honeycomb structure and are attached together by a combination of van der Waals and ionic forces.^[^ ^182^, ^183^ ^]^ Monolayer phosphorene has emerged as an exceptionally promising candidate among various 2D electronic materials due to its bandgap comparable to silicon and high hole mobility.^[^ ^184^, ^185^ ^]^ Phosphorene has a bandgap of ≈2 eV, which can be adjusted by precisely regulating the thickness.^[^ ^186^ ^]^ BP exhibits three crystal structures: orthorhombic, simple cubic, and rhombohedral. Figure 13A,B shows the crystal lattice of orthorhombic BP. A single layer of BP contains two different types of P─P bonds and two different atomic layers. Until now, very few reports have been reported on the electrocatalytic performance of BP, which could be attributed to its inherent challenges, such as poor electrical conductivity and low stability under electrocatalytic conditions. Theoretical study suggested that pure phosphorene exhibits inferior ORR performances due to the adsorption of O* on the phosphorene surface. However, local oxidation can reduce the adsorption strength of O* on the phosphorene and increase the ORR performance.^[^ ^187^ ^]^ In another work, Feng and co‐workers reported that Te doping in BP can enhance the ORR performance of BP.^[^ ^188^ ^]^ Instead of occurring as isolated Te defects, the doped Te atoms tend to form clusters by bonding with each other. Through the synergistic utilization of intrinsic defects and Te dopants, Te cluster's catalytic activity can be precisely adjusted across a broad range. O* exhibit a moderate binding strength at Te sites, further enhances the performance of Te clusters in ORR compared to pristine phosphorene.

Feng and co‐workers first reported a 2D BP‐based metal‐free catalyst for ORR.^[^ ^189^ ^]^ BP was prepared by adopting an electrochemical exfoliation method, whereas g‐C_3_N_4_@CNT (CN) was made by mixing g‐C_3_N_4_ and CNT and annealing at 550°C. A covalently bonded BP with g‐C_3_N_4_@CNT (BP‐CN‐c) was prepared by physically mixing both the materials and heating at 300°C for 2 h under N_2_ atmosphere (Figure 13C). TEM analysis suggested that the CN is uniformly distributed on BP nanosheets, showing abundant heterointerface (Figure 13D,E). Along with the P─P bond, N─P and N─P═O bonds also formed, confirmed by XPS analysis (Figure 13F). The as‐synthesized BP‐CN‐c catalyst showed a similar ORR performance to the commercial Pt/C catalyst (Figure 13G). BP‐CN‐c catalyst showed *E*_1/2_ of 0.84 V versus RHE and *J*_L_ of 5.34 mA cm^‐^^2^, close to Pt/C (0.85 V vs RHE, 5.60 mA cm^‐^^2^). RRDE study revealed that the number of electrons per O_2_ molecule was 3.87–3.92 for BP‐CN‐c catalyst (Figure 13H). The enhanced ORR performance of the BP‐CN‐c catalyst can be ascribed to two main factors: the faster electron transport facilitated by the P─N bonded BP/g‐C_3_N_4_ hybrid and the lower energy barriers for ORR steps taking place on the carbon atoms of g‐C_3_N_4_.

Yu and co‐workers prepared a composite of exfoliated BP (EBP) and amine‐functionalized EBP (N‐EBP), both derived from bulk BP.^[^ ^190^ ^]^ The iso‐type heterostructure (Iso‐EBP) was generated by ultrasonically mixing EBP and N‐EBP in a 1:1 ratio. Iso‐EBP exhibited *E*_onset_ of 0.88 V versus RHE and *J*_L_ of 3 mA cm^‐^^2^, higher compared to EBP and N‐EBP. The electron transfer number of Iso‐EBP was 3.75, which is much higher than EBP (2.92) and N‐EBP (3.18). The amine‐functionalization serves two purposes. First, it accelerates intramolecular electron transfer (P → N) within N‐EBP, generating positively charged P atoms on N‐EBP, which act as efficient active sites for the ORR. Second, it lowers the work function of N‐EBP compared to EBP through chemical modification. Additionally, creating a distinct Iso‐type heterostructure allows directional interfacial electron transfer (N‐EBP → EBP) due to the work function disparity between EBP and N‐EBP. To further improve the activity of iso‐EBP, poly acrylic acid (PAA) was added, which increases the active surface area and shielding effect, increasing stability. Iso‐EBP/PAA exhibited *E*_1/2_ of 0.80 V and *J*_L_ of 5.1 mA cm^‐^^2^, much higher than Iso‐EBP, EBP, and N‐EBP samples. Based on these works, it is clear that a heterostructure formation of BP can enhance the ORR performance of the overall catalyst. Until now, very few studies have been reported on BP as an ORR catalyst. Further study is needed to understand the electrocatalytic properties of BP.

## 5 CHALLENGES AND PERSPECTIVES

This review summarized the recent developments of 2D metal‐free electrocatalysts toward ORR. First, we discussed the reaction mechanism of the ORR process. Since the metal‐free electrocatalysts are composed of carbon, the ORR mechanism could not be explained by the d‐band theory. Instead, the outer sphere electron‐transfer mechanism is suggested, in which the adsorption of O_2_ molecules and the ORR intermediates is preferred on the hetero‐atom doped sites or defective sites. Later, we discussed the metal‐free 2D materials electrocatalysts in two parts; carbon‐based and non‐carbon‐based 2D materials. For each material class, the doping and defect introduction strategies were discussed in detail. First, a systematic description of the ORR catalysts was provided from the viewpoint of 2D material doped with heteroatoms, along with a comprehensive analysis and detailed discussion of the impact of elemental doping on catalytic performances. Then, heterostructure formation and vacancy generation were also discussed for the improvement of the ORR performances.

Despite significant advances in this fledgling field, the study of metal‐free 2D materials has encountered numerous new challenges. First, the production of ultrathin metal‐free 2D materials with high quality and quantity remains a challenging task. Although the strategies for introducing doping and defects have been well‐studied, syntheses that allow quantitative control of the doping level or degree of defects, and syntheses that generate active sites uniformly throughout the 2D materials are very difficult. In addition, existing synthesis methods need to be optimized and scaled to meet the commercial and industrial requirements.

Second, understanding the growth mechanism is vital for the precise design of new 2D electrocatalysts and increasing their intrinsic activity. For example, in the case of 2D carbon materials, the ORR activity depends largely on where the doping elements are substituted. Therefore, understanding the formation of these active sites and being able to selectively generate only those that are favorable to the catalysis would allow us to take synthesis strategies to the next level. To facilitate this understanding, there is a significant need to develop low cost, efficient, and advanced in situ characterization techniques.

Third, most of the metal‐free 2D materials showed similar ORR performance to Pt/C, but were only tested in alkaline solutions. PEMFCs operating in acidic media are a more economical and mature technology.^[^ ^191^ ^]^ Therefore, if the development of acid‐resistant metal‐free 2D materials can be achieved, ORR catalysts in acidic media are expected to be in great demand.

In conclusion, the key strategies for developing new catalysts involve augmenting the number of active centers and enhancing the intrinsic activity of the catalysts. The potential to tailor specific properties for ORR electrocatalytic processes makes developing 2D materials highly promising. Future research will combine experimental electrochemical measurements, theoretical calculations, and advanced spectral characterization as a vital approach for designing and developing novel metal‐free 2D electrocatalysts. The pursuit is to forge 2D electrocatalysts exhibiting exceptional performance and broad utility, aligned with the demands of practical applications. As such, the rapid accomplishments achieved in the realm of metal‐free 2D materials are poised to make substantive contributions to both fundamental research and tangible real‐world implementations in the foreseeable future.

## CONFLICT OF INTEREST STATEMENT

The authors declare no conflicts of interest.

## References

[1] H. Jin , C. Guo , X. Liu , J. Liu , A. Vasileff , Y. Jiao , Y. Zheng , S.‐Z. Qiao , Chem. Rev. 2018, 118, 6337. 29552883 10.1021/acs.chemrev.7b00689

[2] X. Tian , X. F. Lu , B. Y. Xia , X. W. (David) Lou , Joule 2020, 4, 45.

[3] C. F. Shih , T. Zhang , J. Li , C. Bai , Joule 2018, 2, 1925.

[4] M. Chatenet , B. G. Pollet , D. R. Dekel , F. Dionigi , J. Deseure , P. Millet , R. D. Braatz , M. Z. Bazant , M. Eikerling , I. Staffell , P. Balcombe , Y. Shao‐Horn , H. Schäfer , Chem. Soc. Rev. 2022, 51, 4583. 35575644 10.1039/d0cs01079k PMC9332215

[5] Y. Wang , D. F. Ruiz Diaz , K. S. Chen , Z. Wang , X. C. Adroher , Mater. Today 2020, 32, 178.

[6] H.‐F. Wang , Q. Xu , Matter 2019, 1, 565.

[7] H. J. Kim , H. Y. Kim , J. Joo , S. H. Joo , J. S. Lim , J. Lee , H. Huang , M. Shao , J. Hu , J. Y. Kim , B. J. Min , S. W. Lee , M. Kang , K. Lee , S. Choi , Y. Park , Y. Wang , J. Li , Z. Zhang , J. Ma , S.‐I. Choi , J. Mater. Chem. A 2022, 10, 50.

[8] V. R. Stamenkovic , D. Strmcnik , P. P. Lopes , N. M. Markovic , Nat. Mater. 2017, 16, 57. 10.1038/nmat4738 27994237

[9] Y. Sun , S. Gao , F. Lei , Y. Xie , Chem. Soc. Rev. 2015, 44, 623. 25382246 10.1039/c4cs00236a

[10] L. Dai , Y. Xue , L. Qu , H.‐J. Choi , J.‐B. Baek , Chem. Rev. 2015, 115, 4823. 25938707 10.1021/cr5003563

[11] H. Y. Kim , M. Jun , K. Lee , S. H. Joo , ACS Catal. 2023, 13, 355.

[12] H. Y. Kim , M. Jun , S. H. Joo , K. Lee , ACS Nanosci. Au 2023, 3, 28. 37101463 10.1021/acsnanoscienceau.2c00045 PMC10125321

[13] G. M. Tomboc , T. Kim , S. Jung , H. J. Yoon , K. Lee , Small 2022, 18, 2105680. 10.1002/smll.202105680 35102698

[14] Y. Sun , T. Jiang , J. Duan , L. Jiang , X. Hu , H. Zhao , J. Zhu , S. Chen , X. Wang , ACS Catal. 2020, 10, 11371.

[15] Y. Sun , S. Xu , C. A. Ortíz‐Ledón , J. Zhu , S. Chen , J. Duan , Exploration 2021, 1, 20210021. 37323211 10.1002/EXP.20210021 PMC10190981

[16] S. D. Bhoyate , J. Kim , F. M. de Souza , J. Lin , E. Lee , A. Kumar , R. K. Gupta , Coord. Chem. Rev. 2023, 474, 214854.

[17] M. K. Kabiraz , H. J. Kim , Y. Hong , Q. Chang , S.‐I. Choi , Bull. Korean Chem. Soc. 2022, 43, 1093.

[18] M. K. Kabiraz , B. Ruqia , J. Kim , H. Kim , H. J. Kim , Y. Hong , M. J. Kim , Y. K. Kim , C. Kim , W.‐J. Lee , W. Lee , G. H. Hwang , H. C. Ri , H. Baik , H.‐S. Oh , Y. W. Lee , L. Gao , H. Huang , S. M. Paek , Y.‐J. Jo , C. H. Choi , S. W. Han , S.‐I. Choi , ACS Catal. 2022, 12, 3516.

[19] J. Kim , Y. Hong , K. Lee , J. Y. Kim , Adv. Energy Mater. 2020, 10, 2002049.

[20] Y. Jiao , Y. Zheng , M. Jaroniec , S. Z. Qiao , Chem. Soc. Rev. 2015, 44, 2060. 25672249 10.1039/c4cs00470a

[21] Q. Yin , J. M. Tan , C. Besson , Y. V Geletii , D. G. Musaev , A. E. Kuznetsov , Z. Luo , K. I. Hardcastle , C. L. Hill , Science 2010, 328, 342. 20223949 10.1126/science.1185372

[22] M. K. Kabiraz , J. Kim , S.‐I. Choi , Bull. Korean Chem. Soc. 2021, 42, 802.

[23] M. K. Kabiraz , S.‐I. Choi , ChemCatChem 2023, 15, e202300454.

[24] J. Su , R. Ge , K. Jiang , Y. Dong , F. Hao , Z. Tian , G. Chen , L. Chen , Adv. Mater. 2018, 30, 1801351. 10.1002/adma.201801351 29870585

[25] H. Tao , Y. Gao , N. Talreja , F. Guo , J. Texter , C. Yan , Z. Sun , J. Mater. Chem. A 2017, 5, 7257.

[26] Y. Zhang , F. Gao , H. You , Z. Li , B. Zou , Y. Du , Coord. Chem. Rev. 2022, 450, 214244.

[27] P. Lang , N. Yuan , Q. Jiang , Y. Zhang , J. Tang , Energy Technol. 2020, 8, 1900984.

[28] X. Wang , Z. Li , Y. Qu , T. Yuan , W. Wang , Y. Wu , Y. Li , Chem 2019, 5, 1486.

[29] Y. Wang , D. Wang , Y. Li , SmartMat 2021, 2, 56.

[30] Z. W. Seh , J. Kibsgaard , C. F. Dickens , I. Chorkendorff , J. K. Nørskov , T. F. Jaramillo , Science 2017, 355, eaad4998. 28082532 10.1126/science.aad4998

[31] J. Kwag , S. Kim , S. Kang , J. Park , Bull. Korean Chem. Soc. 2023, 44, 488.

[32] J. Cui , Q. Chen , X. Li , S. Zhang , Green Chem. 2021, 23, 6898.

[33] Y.‐L. Zhang , K. Goh , L. Zhao , X.‐L. Sui , X.‐F. Gong , J.‐J. Cai , Q.‐Y. Zhou , H.‐D. Zhang , L. Li , F.‐R. Kong , D.‐M. Gu , Z.‐B. Wang , Nanoscale 2020, 12, 21534. 33112936 10.1039/d0nr05511e

[34] X. Ren , B. Liu , X. Liang , Y. Wang , Q. Lv , A. Liu , J. Electrochem. Soc. 2021, 168, 044521.

[35] C. Zheng , X. Zhang , Z. Zhou , Z. Hu , eScience 2022, 2, 219.

[36] H. Yang , Y. Liu , X. Liu , X. Wang , H. Tian , G. I. N. Waterhouse , P. E. Kruger , S. G. Telfer , S. Ma , eScience 2022, 2, 227.

[37] Y. Wang , J. Wu , S. Tang , J. Yang , C. Ye , J. Chen , Y. Lei , D. Wang , Angew. Chem., Int. Ed. 2023, 62, e202219191. 10.1002/anie.202219191 36808803

[38] K. Gong , F. Du , Z. Xia , M. Durstock , L. Dai , Science 2009, 323, 760. 19197058 10.1126/science.1168049

[39] L. Yang , J. Shui , L. Du , Y. Shao , J. Liu , L. Dai , Z. Hu , Adv. Mater. 2019, 31, 1804799. 10.1002/adma.201804799 30637835

[40] J. Quílez‐Bermejo , E. Morallón , D. Cazorla‐Amorós , Carbon 2020, 165, 434. 10.3390/polym12102382 PMC7602833 33081123

[41] L. Bouleau , S. Pérez‐Rodríguez , J. Quílez‐Bermejo , M. T. Izquierdo , F. Xu , V. Fierro , A. Celzard , Carbon 2022, 189, 349.

[42] H. Singh , S. Zhuang , B. Ingis , B. B. Nunna , E. S. Lee , Carbon 2019, 151, 160.

[43] R. Paul , L. Zhu , H. Chen , J. Qu , L. Dai , Adv. Mater. 2019, 31, 1806403. 10.1002/adma.201806403 30785214

[44] C. Hu , L. Dai , Adv. Mater. 2019, 31, 1804672. 10.1002/adma.201804672 30566275

[45] R. Paul , Q. Dai , C. Hu , L. Dai , Carbon Energy 2019, 1, 19.

[46] W.‐J. Niu , J.‐Z. He , B.‐N. Gu , M.‐C. Liu , Y.‐L. Chueh , Adv. Funct. Mater. 2021, 31, 2103558.

[47] X.‐F. Yang , A. Wang , B. Qiao , J. Li , J. Liu , T. Zhang , Acc. Chem. Res. 2013, 46, 1740. 23815772 10.1021/ar300361m

[48] J. Lili , X. Shuaishuai , X. Baokai , C. Sheng , Z. Junwu , J. Inorg. Mater. 2022, 37, 215.

[49] M. Yu , X. Yuan , J. Guo , N. Tang , S. Ye , J. Liang , L. Jiang , Chemosphere 2021, 284, 131254. 34216926 10.1016/j.chemosphere.2021.131254

[50] Y. Zhao , S. Zhang , R. Shi , G. I. N. Waterhouse , J. Tang , T. Zhang , Mater. Today 2020, 34, 78.

[51] W. Yang , X. Zhang , Y. Xie , Nano Today 2016, 11, 793.

[52] D. Chen , Y. Zou , S. Wang , Mater. Today Energy 2019, 12, 250.

[53] Z. Li , Y. Chen , T. Ma , Y. Jiang , J. Chen , H. Pan , W. Sun , Adv. Energy Mater. 2021, 11, 2101202.

[54] K. Yuan , D. Lützenkirchen‐Hecht , L. Li , L. Shuai , Y. Li , R. Cao , M. Qiu , X. Zhuang , M. K. H. Leung , Y. Chen , U. Scherf , J. Am. Chem. Soc. 2020, 142, 2404. 31902210 10.1021/jacs.9b11852

[55] S. Zhou , X. Yang , X. Xu , S. X. Dou , Y. Du , J. Zhao , J. Am. Chem. Soc. 2020, 142, 308. 31840999 10.1021/jacs.9b10588

[56] Y. Wu , J. Cai , Y. Xie , S. Niu , Y. Zang , S. Wu , Y. Liu , Z. Lu , Y. Fang , Y. Guan , X. Zheng , J. Zhu , X. Liu , G. Wang , Y. Qian , Adv. Mater. 2020, 32, 1904346. 10.1002/adma.201904346 32449199

[57] X. Zheng , P. Cui , Y. Qian , G. Zhao , X. Zheng , X. Xu , Z. Cheng , Y. Liu , S. X. Dou , W. Sun , Angew. Chem., Int. Ed. 2020, 59, 14533. 10.1002/anie.202005241 32485085

[58] J. Kim , Y. Yang , A. Seong , H.‐J. Noh , C. Kim , S. Joo , A. Cho , L. Zhang , J. Zhou , J.‐Q. Wang , J. W. Han , J. Mahmood , J.‐B. Baek , G. Kim , J. Mater. Chem. A 2020, 8, 14927.

[59] C. Liu , A. Tian , Q. Li , T. Wang , G. Qin , S. Li , C. Sun , Adv. Funct. Mater. 2023, 33, 2210759.

[60] D. Deng , L. Yu , X. Pan , S. Wang , X. Chen , P. Hu , L. Sun , X. Bao , Chem. Commun. 2011, 47, 10016. 10.1039/c1cc13033a 21837331

[61] X.‐F. Li , Q.‐K. Li , J. Cheng , L. Liu , Q. Yan , Y. Wu , X.‐H. Zhang , Z.‐Y. Wang , Q. Qiu , Y. Luo , J. Am. Chem. Soc. 2016, 138, 8706. 27383680 10.1021/jacs.6b04778

[62] R. Ma , G. Lin , Y. Zhou , Q. Liu , T. Zhang , G. Shan , M. Yang , J. Wang , npj Comput. Mater. 2019, 5, 78.

[63] X. Ge , A. Sumboja , D. Wuu , T. An , B. Li , F. W. T. Goh , T. S. A. Hor , Y. Zong , Z. Liu , ACS Catal. 2015, 5, 4643.

[64] T. Kwon , Y. Lim , J. Cho , R. Lawler , B. J. Min , W. A. Goddard , S. S. Jang , J. Y. Kim , Mater. Today 2022, 58, 135.

[65] M. Zatoń , J. Rozière , D. J. Jones , Sustainable Energy Fuels 2017, 1, 409.

[66] J. Woo , J. S. Lim , J. H. Kim , S. H. Joo , Chem. Commun. 2021, 57, 7350. 10.1039/d1cc02667d 34231572

[67] A. Kulkarni , S. Siahrostami , A. Patel , J. K. Nørskov , Chem. Rev. 2018, 118, 2302. 29405702 10.1021/acs.chemrev.7b00488

[68] S. Sui , X. Wang , X. Zhou , Y. Su , S. Riffat , C. Liu , J. Mater. Chem. A 2017, 5, 1808.

[69] J. K. Nørskov , J. Rossmeisl , A. Logadottir , L. Lindqvist , J. R. Kitchin , T. Bligaard , H. Jónsson , J. Phys. Chem. B 2004, 108, 17886. 10.1021/jp047349j 39682080

[70] J. H. Montoya , L. C. Seitz , P. Chakthranont , A. Vojvodic , T. F. Jaramillo , J. K. Nørskov , Nat. Mater. 2017, 16, 70. 10.1038/nmat4778 27994241

[71] X. Guo , S. Lin , J. Gu , S. Zhang , Z. Chen , S. Huang , ACS Catal. 2019, 9, 11042.

[72] Y. Meng , X. Huang , H. Lin , P. Zhang , Q. Gao , W. Li , Front. Chem. 2019, 7, 759. 31781542 10.3389/fchem.2019.00759 PMC6861163

[73] Y. Li , Y. Tong , F. Peng , J. Energy Chem. 2020, 48, 308.

[74] V. Gueskine , M. Vagin , M. Berggren , X. Crispin , I. Zozoulenko , Electrochem. Sci. Adv. 2023, 3, e2100191.

[75] N. Ramaswamy , S. Mukerjee , J. Phys. Chem. C 2011, 115, 18015.

[76] K. Wan , Z. Yu , X. Li , M. Liu , G. Yang , J. Piao , Z. Liang , ACS Catal. 2015, 5, 4325.

[77] C. H. Choi , H.‐K. Lim , M. W. Chung , J. C. Park , H. Shin , H. Kim , S. I. Woo , J. Am. Chem. Soc. 2014, 136, 9070. 24905892 10.1021/ja5033474

[78] N. Ramaswamy , S. Mukerjee , Adv. Phys. Chem. 2012, 2012, 491604.

[79] L. Yu , X. Pan , X. Cao , P. Hu , X. Bao , J. Catal. 2011, 282, 183.

[80] X. Zhou , J. Qiao , L. Yang , J. Zhang , Adv. Energy Mater. 2014, 4, 1301523.

[81] D. Li , Y. Jia , G. Chang , J. Chen , H. Liu , J. Wang , Y. Hu , Y. Xia , D. Yang , X. Yao , Chem 2018, 4, 2345.

[82] J. Ortiz‐Medina , Z. Wang , R. Cruz‐Silva , A. Morelos‐Gomez , F. Wang , X. Yao , M. Terrones , M. Endo , Adv. Mater. 2019, 31, 1805717. 10.1002/adma.201805717 30687977

[83] K. Choi , S. Kim , ACS Nano 2022, 16, 16394. 36219762 10.1021/acsnano.2c05607

[84] K.‐H. Wu , D.‐W. Wang , D.‐S. Su , I. R. Gentle , ChemSusChem 2015, 8, 2772. 26334773 10.1002/cssc.201500373

[85] Y. Ji , H. Dong , C. Liu , Y. Li , J. Mater. Chem. A 2018, 6, 13489.

[86] F. An , X. Bao , X. Deng , Z. Ma , X. Wang , New Carbon Mater. 2022, 37, 338.

[87] X. Liu , L. Dai , Nat. Rev. Mater. 2016, 1, 16064.

[88] Y. Jiao , Y. Zheng , K. Davey , S.‐Z. Qiao , Nat. Energy 2016, 1, 16130.

[89] M. Zhang , L. Dai , Nano Energy 2012, 1, 514.

[90] D. Usachov , O. Vilkov , A. Grüneis , D. Haberer , A. Fedorov , V. K. Adamchuk , A. B. Preobrajenski , P. Dudin , A. Barinov , M. Oehzelt , C. Laubschat , D. V. Vyalikh , Nano Lett. 2011, 11, 5401. 22077830 10.1021/nl2031037

[91] M. Yang , L. Zhou , J. Wang , Z. Liu , Z. Liu , J. Phys. Chem. C 2012, 116, 844.

[92] Y. Shao , Z. Jiang , Q. Zhang , J. Guan , ChemSusChem 2019, 12, 2133. 30806034 10.1002/cssc.201900060

[93] Z. Hou , X. Wang , T. Ikeda , K. Terakura , M. Oshima , M. Kakimoto , Phys. Rev. B 2013, 87, 165401. 10.1021/jp307405r 23270514

[94] L. Zhao , R. He , K. T. Rim , T. Schiros , K. S. Kim , H. Zhou , C. Gutiérrez , S. P. Chockalingam , C. J. Arguello , L. Pálová , D. Nordlund , M. S. Hybertsen , D. R. Reichman , T. F. Heinz , P. Kim , A. Pinczuk , G. W. Flynn , A. N. Pasupathy , Science 2011, 333, 999. 21852495 10.1126/science.1208759

[95] T. Schiros , D. Nordlund , L. Pálová , D. Prezzi , L. Zhao , K. S. Kim , U. Wurstbauer , C. Gutiérrez , D. Delongchamp , C. Jaye , D. Fischer , H. Ogasawara , L. G. M. Pettersson , D. R. Reichman , P. Kim , M. S. Hybertsen , A. N. Pasupathy , Nano Lett. 2012, 12, 4025. 22746249 10.1021/nl301409h

[96] R. A. Sidik , A. B. Anderson , N. P. Subramanian , S. P. Kumaraguru , B. N. Popov , J. Phys. Chem. B 2006, 110, 1787. 16471746 10.1021/jp055150g

[97] H. Niwa , K. Horiba , Y. Harada , M. Oshima , T. Ikeda , K. Terakura , J. Ozaki , S. Miyata , J. Power Sources 2009, 187, 93.

[98] S.‐F. Huang , K. Terakura , T. Ozaki , T. Ikeda , M. Boero , M. Oshima , J. Ozaki , S. Miyata , Phys. Rev. B 2009, 80, 235410.

[99] G. Lemes , D. Sebastián , E. Pastor , M. J. Lázaro , J. Power Sources 2019, 438, 227036.

[100] J. Guo , S. Zhang , M. Zheng , J. Tang , L. Liu , J. Chen , X. Wang , Int. J. Hydrogen Energy 2020, 45, 32402.

[101] L. Lai , J. R. Potts , D. Zhan , L. Wang , C. K. Poh , C. Tang , H. Gong , Z. Shen , J. Lin , R. S. Ruoff , Energy Environ. Sci. 2012, 5, 7936.

[102] J. H. Dumont , U. Martinez , K. Artyushkova , G. M. Purdy , A. M. Dattelbaum , P. Zelenay , A. Mohite , P. Atanassov , G. Gupta , ACS Appl. Nano Mater. 2019, 2, 1675.

[103] Q. Xiang , Y. Liu , X. Zou , B. Hu , Y. Qiang , D. Yu , W. Yin , C. Chen , ACS Appl. Mater. Interfaces 2018, 10, 10842. 29547254 10.1021/acsami.7b19122

[104] L. Wang , H. Dong , Z. Guo , L. Zhang , T. Hou , Y. Li , J. Phys. Chem. C 2016, 120, 17427.

[105] G. Fazio , L. Ferrighi , C. Di Valentin , J. Catal. 2014, 318, 203.

[106] S. Agnoli , M. Favaro , J. Mater. Chem. A 2016, 4, 5002.

[107] L. Yang , S. Jiang , Y. Zhao , L. Zhu , S. Chen , X. Wang , Q. Wu , J. Ma , Y. Ma , Z. Hu , Angew. Chem., Int. Ed. 2011, 50, 7132. 10.1002/anie.201101287 21688363

[108] A. B. Jorge , R. Jervis , A. P. Periasamy , M. Qiao , J. Feng , L. N. Tran , M.‐M. Titirici , Adv. Energy Mater. 2020, 10, 1902494.

[109] M. Shao , Q. Chang , J.‐P. Dodelet , R. Chenitz , Chem. Rev. 2016, 116, 3594. 26886420 10.1021/acs.chemrev.5b00462

[110] K. Kakaei , M. D. Esrafili , A. Ehsani , in Graphene Surfaces: particles and catalyst. Elsevier, Amsterdam 2019, 203.

[111] L. Qin , L. Wang , X. Yang , R. Ding , Z. Zheng , X. Chen , B. Lv , J. Catal. 2018, 359, 242.

[112] J. P. V. Tafoya , S. Doszczeczko , M. M. Titirici , A. B. J. Sobrido , Int. J. Hydrogen Energy 2022, 47, 5462.

[113] Z. Yao , M. Hu , Z. Iqbal , X. Wang , ACS Catal. 2020, 10, 160.

[114] Y. Zheng , Y. Jiao , L. Ge , M. Jaroniec , S. Z. Qiao , Angew. Chem., Int. Ed. 2013, 52, 3110. 10.1002/anie.201209548 23341193

[115] I. T. Kim , M. J. Song , Y. B. Kim , M. W. Shin , Int. J. Hydrogen Energy 2016, 41, 22026.

[116] M. Zhang , H. Tao , Y. Liu , C. Yan , S. Hong , J. Masa , A. W. Robertson , S. Liu , J. Qiu , Z. Sun , ACS Sustainable Chem. Eng. 2019, 7, 3434.

[117] F. Banhart , J. Kotakoski , A. V Krasheninnikov , ACS Nano 2011, 5, 26. 21090760 10.1021/nn102598m

[118] C. Tang , Q. Zhang , Adv. Mater. 2017, 29, 1604103. 10.1002/adma.201604103 28067956

[119] H. Jiang , J. Gu , X. Zheng , M. Liu , X. Qiu , L. Wang , W. Li , Z. Chen , X. Ji , J. Li , Energy Environ. Sci. 2019, 12, 322.

[120] Q. Wang , Y. Ji , Y. Lei , Y. Wang , Y. Wang , Y. Li , S. Wang , ACS Energy Lett. 2018, 3, 1183.

[121] Y. Gong , L. Shen , Z. Kang , K. Liu , Q. Du , D. Ye , H. Zhao , X. A. Sun , J. Zhang , J. Mater. Chem. A 2020, 8, 21408.

[122] R. Liu , X. Gao , J. Zhou , H. Xu , Z. Li , S. Zhang , Z. Xie , J. Zhang , Z. Liu , Adv. Mater. 2017, 29, 1604665. 10.1002/adma.201604665 28251693

[123] R. Matsuoka , R. Sakamoto , K. Hoshiko , S. Sasaki , H. Masunaga , K. Nagashio , H. Nishihara , J. Am. Chem. Soc. 2017, 139, 3145. 28199105 10.1021/jacs.6b12776

[124] R. Liu , H. Liu , Y. Li , Y. Yi , X. Shang , S. Zhang , X. Yu , S. Zhang , H. Cao , G. Zhang , Nanoscale 2014, 6, 11336. 25141067 10.1039/c4nr03185g

[125] H. Yu , Y. Xue , Y. Li , Adv. Mater. 2019, 31, 1803101. 10.1002/adma.201803101 31119816

[126] S. Zhang , Y. Cai , H. He , Y. Zhang , R. Liu , H. Cao , M. Wang , J. Liu , G. Zhang , Y. Li , H. Liu , B. Li , J. Mater. Chem. A 2016, 4, 4738.

[127] Y. Wang , X. Jiang , ACS Appl. Mater. Interfaces 2013, 5, 11597. 24187942 10.1021/am402669y

[128] M. Rahsepar , M. R. Nobakht , H. Kim , M. Pakshir , Appl. Surf. Sci. 2018, 447, 182.

[129] M. Skorupska , A. Ilnicka , J. P. Lukaszewicz , Sci. Rep. 2021, 11, 23970. 34907258 10.1038/s41598-021-03403-8 PMC8671485

[130] C. Ou , H. Chen , H. Wang , Y. Liao , R. Li , H. Liu , Electrochim. Acta 2021, 380, 138256.

[131] X. Lu , D. Wang , L. Ge , L. Xiao , H. Zhang , L. Liu , J. Zhang , M. An , P. Yang , New J. Chem. 2018, 42, 19665.

[132] J. Lee , S. Noh , N. D. Pham , J. H. Shim , Electrochim. Acta 2019, 313, 1.

[133] K. Kakaei , A. Balavandi , J. Colloid Interface Sci. 2017, 490, 819. 27997850 10.1016/j.jcis.2016.12.011

[134] X. Zhang , X. Wen , C. Pan , X. Xiang , C. Hao , Q. Meng , Z. Q. Tian , P. K. Shen , S. P. Jiang , Chem. Eng. J. 2022, 431, 133216.

[135] E. A. A. Nazer , A. Muthukrishnan , Catal. Sci. Technol. 2020, 10, 6659.

[136] Y. Zhou , Y. Sun , H. Wang , C. Zhu , J. Gao , D. Wu , H. Huang , Y. Liu , Z. Kang , Inorg. Chem. Front. 2018, 5, 2985.

[137] Y. Liao , H. Chen , C. Ou , L. Bao , R. Li , H. Liu , J. Electroanal. Chem. 2022, 921, 116560.

[138] C. Cheng , Y. Li , C. Maouche , B. Li , Y. Zhou , S. Wang , X. Cheng , J. Yang , J. Electroanal. Chem. 2021, 883, 115058.

[139] J. Huang , J. Han , T. Gao , X. Zhang , J. Li , Z. Li , P. Xu , B. Song , Carbon 2017, 124, 34.

[140] J. Liu , L. Wei , H. Wang , G. Lan , H. Yang , J. Shen , Electrochim. Acta 2020, 364, 137335.

[141] G. Ren , B. Huang , C. Li , C. Lin , Y. Qian , J. Electroanal. Chem. 2020, 877, 114732.

[142] M. Qiao , C. Tang , G. He , K. Qiu , R. Binions , I. P. Parkin , Q. Zhang , Z. Guo , M. M. Titirici , J. Mater. Chem. A 2016, 4, 12658.

[143] Q. Li , D. Xu , X. Ou , F. Yan , Chem. Asian J. 2017, 12, 1816. 28493381 10.1002/asia.201700586

[144] J. Li , Y. Zhang , X. Zhang , J. Huang , J. Han , Z. Zhang , X. Han , P. Xu , B. Song , ACS Appl. Mater. Interfaces 2017, 9, 398. 27983785 10.1021/acsami.6b12547

[145] Y. Zhao , J. Wan , H. Yao , L. Zhang , K. Lin , L. Wang , N. Yang , D. Liu , L. Song , J. Zhu , L. Gu , L. Liu , H. Zhao , Y. Li , D. Wang , Nat. Chem. 2018, 10, 924. 30082882 10.1038/s41557-018-0100-1

[146] Q. Lv , W. Si , Z. Yang , N. Wang , Z. Tu , Y. Yi , C. Huang , L. Jiang , M. Zhang , J. He , Y. Long , ACS Appl. Mater. Interfaces 2017, 9, 29744. 28812362 10.1021/acsami.7b08115

[147] M. Li , K. Wang , Q. Lv , Chem. Res. Chin. Univ. 2021, 37, 1283. 10.1007/s40242-021-1197-0 PMC8339700 34376961

[148] F. Besharat , F. Ahmadpoor , Z. Nezafat , M. Nasrollahzadeh , N. R. Manwar , P. Fornasiero , M. B. Gawande , ACS Catal. 2022, 12, 5605.

[149] Y. Zheng , J. Liu , J. Liang , M. Jaroniec , S. Z. Qiao , Energy Environ. Sci. 2012, 5, 6717.

[150] Y. Zheng , Y. Jiao , J. Chen , J. Liu , J. Liang , A. Du , W. Zhang , Z. Zhu , S. C. Smith , M. Jaroniec , G. Q. (Max) Lu , S. Z. Qiao , J. Am. Chem. Soc. 2011, 133, 20116. 22082332 10.1021/ja209206c

[151] J. Liang , Y. Zheng , J. Chen , J. Liu , D. Hulicova‐Jurcakova , M. Jaroniec , S. Z. Qiao , Angew. Chem., Int. Ed. 2012, 51, 3892. 10.1002/anie.201107981 22389097

[152] X. Fu , X. Hu , Z. Yan , K. Lei , F. Li , F. Cheng , J. Chen , Chem. Commun. 2016, 52, 1725. 10.1039/c5cc08897f 26666314

[153] Q. Li , S. Song , Z. Mo , L. Zhang , Y. Qian , C. Ge , Appl. Surf. Sci. 2022, 579, 152006.

[154] A. K. Mrinalini Kalyani , R. Rajeev , L. Benny , A. R. Cherian , A. Varghese , Mater. Today Chem. 2023, 30, 101523.

[155] T. Suter , V. Brázdová , K. McColl , T. S. Miller , H. Nagashima , E. Salvadori , A. Sella , C. A. Howard , C. W. M. Kay , F. Corà , P. F. McMillan , J. Phys. Chem. C 2018, 122, 25183.

[156] X. Wang , L. Wang , F. Zhao , C. Hu , Y. Zhao , Z. Zhang , S. Chen , G. Shi , L. Qu , Nanoscale 2015, 7, 3035. 25603736 10.1039/c4nr05343e

[157] I. Y. Kim , S. Kim , S. Premkumar , J.‐H. Yang , S. Umapathy , A. Vinu , Small 2020, 16, 1903572. 10.1002/smll.201903572 31782908

[158] M. Wang , Z. Wu , L. Dai , J. Electroanal. Chem. 2015, 753, 16.

[159] P. Selvarajan , M. Fawaz , C. I. Sathish , M. Li , D. Chu , X. Yu , M. B. H. Breesec , J. Yi , A. Vinu , Adv. Energy Sustainable Res. 2021, 2, 2100104.

[160] J. L. Garcia , T. Miyao , J. Inukai , B. J. V. Tongol , Mater. Chem. Phys. 2022, 288, 126415.

[161] C. Li , X. Li , X. Zhang , X. Yang , L. Wang , W. Lü , J. Electrochem. Soc. 2020, 167, 100534.

[162] C. Xu , Q. Han , Y. Zhao , L. Wang , Y. Li , L. Qu , J. Mater. Chem. A 2015, 3, 1841.

[163] Z. Pei , J. Zhao , Y. Huang , Y. Huang , M. Zhu , Z. Wang , Z. Chen , C. Zhi , J. Mater. Chem. A 2016, 4, 12205.

[164] S. Roy , X. Zhang , A. B. Puthirath , A. Meiyazhagan , S. Bhattacharyya , M. M. Rahman , G. Babu , S. Susarla , S. K. Saju , M. K. Tran , L. M. Sassi , M. A. S. R. Saadi , J. Lai , O. Sahin , S. M. Sajadi , B. Dharmarajan , D. Salpekar , N. Chakingal , A. Baburaj , X. Shuai , A. Adumbumkulath , K. A. Miller , J. M. Gayle , A. Ajnsztajn , T. Prasankumar , V. V. J. Harikrishnan , V. Ojha , H. Kannan , A. Z. Khater , Z. Zhu , S. A. Iyengar, P. A. d. S. Autreto, E. F. Oliveira, G. Gao, A. G. Birdwell, M. R. Neupane, T. G. Ivanov, J. Taha‐Tijerina, R. M. Yadav, S. Arepalli, R. Vajtai, P. M. Ajayan, Adv. Mater. 2021, 33, 2101589. 10.1002/adma.202101589 34561916

[165] M. Rafiq , X. Hu , Z. Ye , A. Qayum , H. Xia , L. Hu , F. Lu , P. K. Chu , Nano Energy 2022, 91, 106661.

[166] A. F. Khan , E. P. Randviir , D. A. C. Brownson , X. Ji , G. C. Smith , C. E. Banks , Electroanalysis 2017, 29, 622.

[167] P. Marbaniang , I. Patil , M. Lokanathan , H. Parse , D. Catherin Sesu , S. Ingavale , B. Kakade , ACS Sustainable Chem. Eng. 2018, 6, 11115.

[168] I. M. Patil , M. Lokanathan , B. Kakade , J. Mater. Chem. A 2016, 4, 4506.

[169] I. M. Patil , C. P. Jijil , M. Lokanathan , A. Swami , B. Kakade , Sustainable Energy Fuels 2018, 2, 252.

[170] I. M. Patil , M. Lokanathan , B. Ganesan , A. Swami , B. Kakade , Chem. Eur. J. 2017, 23, 676. 27709715 10.1002/chem.201604231

[171] R. Kumar , K. Gopalakrishnan , I. Ahmad , C. N. R. Rao , Adv. Funct. Mater. 2015, 25, 5910.

[172] M. Fan , Z. Wang , Y. Zhao , Q. Yuan , J. Cui , J. Raj , K. Sun , A. Wang , J. Wu , H. Sun , B. Li , L. Wang , J. Jiang , Carbon Energy 2023, 5, e309.

[173] S. Thomas , M. S. Manju , K. M. Ajith , S. U. Lee , M. Asle Zaeem , Phys. E 2020, 123, 114180.

[174] Y. K. Recepoglu , A. Y. Goren , V. Vatanpour , Y. Yoon , A. Khataee , Desalination 2022, 533, 115782.

[175] J. Wang , J. Hao , D. Liu , S. Qin , D. Portehault , Y. Li , Y. Chen , W. Lei , ACS Energy Lett. 2017, 2, 306.

[176] S. Angizi , M. A. Akbar , M. Darestani‐Farahani , P. Kruse , ECS J. Solid State Sci. Technol. 2020, 9, 83004.

[177] M. A. Ahsan , T. He , K. Eid , A. M. Abdullah , M. L. Curry , A. Du , A. R. Puente Santiago , L. Echegoyen , J. C. Noveron , J. Am. Chem. Soc. 2021, 143, 1203. 33401899 10.1021/jacs.0c12386

[178] P. Marbaniang , S. Ingavale , D. Catherin , N. Ramgir , A. Swami , B. Kakade , J. Catal. 2019, 378, 104.

[179] L. Qu , Z. Zhang , H. Zhang , H. Zhang , S. Dong , Appl. Surf. Sci. 2018, 448, 618.

[180] M. Koleśnik‐Gray , L. Meingast , M. Siebert , T. Unbehaun , T. Huf , G. Ellrott , G. Abellán , S. Wild , V. Lloret , U. Mundloch , J. Schwarz , M. Niebauer , M. Szabo , M. Rommel , A. Hutzler , F. Hauke , A. Hirsch , V. Krstić , npj 2D Mater. Appl. 2023, 7, 21.

[181] F. Xia , H. Wang , J. C. M. Hwang , A. H. C. Neto , L. Yang , Nat. Rev. Phys. 2019, 1, 306.

[182] J. Qiao , X. Kong , Z.‐X. Hu , F. Yang , W. Ji , Nat. Commun. 2014, 5, 4475. 25042376 10.1038/ncomms5475 PMC4109013

[183] L. Shulenburger , A. D. Baczewski , Z. Zhu , J. Guan , D. Tománek , Nano Lett. 2015, 15, 8170. 26523860 10.1021/acs.nanolett.5b03615

[184] J. Xiao , M. Long , X. Zhang , J. Ouyang , H. Xu , Y. Gao , Sci. Rep. 2015, 5, 9961. 26035176 10.1038/srep09961 PMC4451805

[185] G. Long , D. Maryenko , J. Shen , S. Xu , J. Hou , Z. Wu , W. K. Wong , T. Han , J. Lin , Y. Cai , R. Lortz , N. Wang , Nano Lett. 2016, 16, 7768. 27960491 10.1021/acs.nanolett.6b03951

[186] X. Ling , H. Wang , S. Huang , F. Xia , M. S. Dresselhaus , Proc. Natl. Acad. Sci. U. S. A. 2015, 112, 4523. 25820173 10.1073/pnas.1416581112 PMC4403146

[187] X.‐X. Xue , S. Shen , X. Jiang , P. Sengdala , K. Chen , Y. Feng , J. Phys. Chem. Lett. 2019, 10, 3440. 31181929 10.1021/acs.jpclett.9b00891

[188] J. Zhu , X. Jiang , Y. Yang , Q. Chen , X.‐X. Xue , K. Chen , Y. Feng , Phys. Chem. Chem. Phys. 2019, 21, 22939. 31598612 10.1039/c9cp04164h

[189] X. Wang , R. K. M. Raghupathy , C. J. Querebillo , Z. Liao , D. Li , K. Lin , M. Hantusch , Z. Sofer , B. Li , E. Zschech , I. M. Weidinger , T. D. Kühne , H. Mirhosseini , M. Yu , X. Feng , Adv. Mater. 2021, 33, 2008752. 10.1002/adma.202008752 PMC11469023 33939200

[190] Z. Yuan , J. Li , Z. Fang , M. Yang , K. Mai , D. Yu , J. Energy Chem. 2021, 63, 468.

[191] H. Zhang , P. K. Shen , Chem. Rev. 2012, 112, 2780. 22339373 10.1021/cr200035s

